# First demonstration of effective spatial training for near transfer to spatial performance and far transfer to a range of mathematics skills at 8 years

**DOI:** 10.1111/desc.12909

**Published:** 2019-11-06

**Authors:** Katie A. Gilligan, Michael S. C. Thomas, Emily K. Farran

**Affiliations:** ^1^ School of Psychology University of Surrey Guilford UK; ^2^ Department of Psychology and Human Development UCL Institute of Education University College London London UK; ^3^ Department of Psychological Sciences Birkbeck, University of London London UK

**Keywords:** cognitive training, explicit instruction, mathematics, mental rotation, spatial scaling, transfer

## Abstract

There is evidence that spatial thinking is malleable, and that spatial and mathematical skills are associated (Mix et al. [2016] *Journal of Experimental Psychology: General*, 145, 1206; Mix et al. [2017] *Journal of Cognition and Development*, 18, 465; Uttal et al. [2013] *Psychological Bulletin*, 139, 352). However, few studies have investigated transfer of spatial training gains to mathematics outcomes in children, and no known studies have compared different modes of spatial instruction (explicit vs. implicit instruction). Based on a sample of 250 participants, this study compared the effectiveness of explicit and implicit spatial instruction in eliciting near transfer (to the specific spatial skills trained), intermediate transfer (to untrained spatial skills) and far transfer (to mathematics domains) at age 8. Spatial scaling and mental rotation skills were chosen as training targets as previous studies have found, and proposed explanations for, associations between these skills and mathematics in children of this age (*Journal of Experimental Psychology: General*, 145, 2016 and 1206). In this study, spatial training led to near, intermediate and far transfer of gains. Mental visualization and proportional reasoning were proposed to explain far transfer from mental rotation and spatial scaling skills respectively. For most outcomes, except for geometry, there was no difference in the effectiveness of implicit (practice with feedback) compared to explicit instruction (instructional videos). From a theoretical perspective, the study identified a specific causal effect of spatial skills on mathematics skills in children. Practically, the results also highlight the potential of instructional videos as a method of introducing spatial thinking into the classroom.

## Graphical abstract

Both explicit and implicit training of mental rotation and spatial scaling led to near transfer (to the specific spatial skills trained), intermediate transfer (to untrained spatial skills) and far transfer (to mathematics domains) in children aged 8 years. Mental visualization is proposed to explain far transfer of gains from mental rotation to missing term problems and geometry performance. Proportional reasoning is proposed to explain far transfer of gains from spatial scaling to number line estimation and geometry performance. This study identified a specific causal effect of spatial skills on mathematics skills in children (*n* = 250).

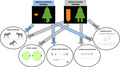

**Research Highlights**

- Both explicit instruction (instructional videos) and implicit instruction (task practice with feedback) elicited gains in spatial performance at 8 years.
- Training spatial skills led to near, intermediate and far transfer of gains, even after controlling for expectation and engagement effects.
- Mental visualization and proportional reasoning were proposed to explain far transfer from mental rotation and spatial scaling skills, to mathematics respectively.
- The transfer of spatial training gains from spatial to mathematics sub‐domains provides evidence for a causal influence of spatial thinking on mathematics performance.

## 1 INTRODUCTION

### 1.1 Rationale

This paper investigates the impact of spatial training on spatial skills that have been specifically trained (near transfer), non‐trained spatial skills (intermediate transfer) and mathematics skills (far transfer). That spatial training interventions can improve mathematical ability in children is supported by evidence that spatial ability is malleable, and that there are significant associations between mathematics and spatial skills in childhood populations. In a meta‐analysis of 217 studies, Uttal et al. (2013) reported an effect size of almost one half a standard deviation for training studies that compared spatial training to control conditions (Hedges *G* = 0.47). The effect size increased to 0.61 (Hedges *G*) when the analysis was limited to studies of children under 13 years, demonstrating the particular malleability of spatial thinking in childhood (*N* = 53 studies). Note that similarly to Cohen's *d*, Hedges *G* values of 0.2, 0.5 and 0.8 correspond to small, medium and large effects respectively (Cohen, 1988). There is also convincing evidence that spatial and mathematical thinking are associated longitudinally in childhood. For example, spatial thinking measured using the Test of Spatial Assembly [TOSA]) at 3 years predicts 27% of the variation in mathematics problem solving at 5 years (Verdine et al., 2014), and pattern construction skills at 5 years explain approximately 9% of the variation in mathematics performance at 7 years (Gilligan, Flouri, & Farran, 2017).

However, the literature does not support a simple linear coupling between all aspects of spatial and mathematical cognition (Fias & Bonato, 2018). There is evidence that spatial‐mathematical relations are specific to certain spatial and mathematics tasks and that these relations may differ across development. Gilligan, Hodgkiss, Thomas, and Farran (2018) measured the relationship between four different spatial sub‐domains and mathematics. They found that spatial scaling (or the ability to transform distance information from one representation to another representation of a different size; Frick & Newcombe, 2012) was the strongest spatial predictor of standardized mathematics performance in 6–10 year olds when compared to perspective taking, disembedding and mental rotation. Mental rotation had an age‐dependent role for 6–8 year olds only (Gilligan et al., 2018). Similar age‐dependent findings were reported by Mix et al. (2016, 2017) who found that mental rotation was a significant predictor of mathematics performance at 6 and 9 years but not at 11 years. Frick (2019) also reported that, in comparison to other spatial skills (diagrammatic representation, cross‐sectioning, mental transformation and perspective taking), spatial scaling and mental rotation at 6.5 years explained at least 24% of the variation in mathematics performance at 8.5 years. This included both arithmetic items and items assessing numeric‐logical and spatial functions (e.g. number sequences, counting magnitudes, counting cubes, estimating line lengths; Frick, 2019). Taken together, the selection of spatial sub‐domains for training studies should reflect the facts that (a) not all spatial skills are equally associated with all mathematics outcomes and (b) spatial‐mathematical associations are developmentally sensitive.

Mental rotation and spatial scaling were targeted for training in this study. As outlined, these skills have previously been associated with mathematics achievement in children aged 6–9 years. Furthermore, underlying cognitive mechanisms have been proposed that may explain associations between these spatial skills and mathematics outcomes (e.g. Gilligan et al., 2018; Mix et al., 2016, 2017). These proposed underlying mechanisms influenced not only the selection of training targets, but also the selection of mathematics measures for inclusion in this study. Specifically, mental rotation is proposed to elicit active processing, including mental visualization and manipulation of objects (Lourenco, Cheung, & Aulet, 2018; Mix et al., 2016). Thus, mental rotation training may have benefits for mathematics tasks requiring the mental manipulation or organization of numbers, for example, complex mathematical word problems or multidigit calculations (Lourenco et al., 2018). Missing term problems were included in the task battery of this study as mathematics tasks of this type require mental manipulation of numbers. In contrast, spatial scaling is proposed to elicit intensive quantification skills (proportional reasoning). Thus, spatial scaling training may improve performance on mathematics tasks that require proportional reasoning, for example, number line estimation and geometry performance (Newcombe, Levine, & Mix, 2015; Newcombe, Möhring, & Frick, 2018; Rouder & Geary, 2014). For this reason, both number line and Geometry Tasks were included in the task battery of this study.

This study included participants aged approximately 8 years. As outlined above, there is evidence of significant spatial‐mathematics relations at this age. Furthermore, as described in the next section, this age range overlapped with other spatial training studies that investigated transfer of gains to mathematics (Cheng & Mix, 2014; Hawes, Moss, Caswell, & Poliszczuk, 2015). Thus, the inclusion of participants aged 8 years allowed for meaningful comparisons between this, and previous studies. Additionally, children of this age were deemed old enough for independent computer‐based training.

### 1.2 Evidence of transfer of spatial training gains to mathematics

Spatial interventions that integrate spatial thinking into mathematical instruction report gains in both spatial (near and intermediate transfer) and mathematical outcomes (far transfer; Hawes, Moss, Caswell, Naqvi, & MacKinnon, 2017; Lowrie, Logan, & Ramful, 2017). However, these studies cannot offer insight into the underlying causal relationship between spatial and mathematical domains, as it is not possible to disentangle the impact of the spatial, and mathematical aspects of training respectively. Few studies have investigated transfer of gains from spatial training (with no mathematical component) to mathematics. Cheng and Mix (2014) reported significant gains in mental rotation (near transfer) and mathematical calculation (far transfer) following 40‐min of mental rotation training in 6–8 year olds, compared to a control group. Gains were specific to missing term arithmetic problems, for example, 4 + __ = 9. In a similar mental rotation training study of 6–8 year olds, Hawes et al. (2015) failed to replicate these findings with respect to far transfer. Improvements in mental rotation (near transfer) and mental transformation (intermediate transfer) were reported for the training group who completed 15 sessions of computerized mental rotation training, compared to controls. However, no improvements in mathematics skills including non‐verbal arithmetic or missing term arithmetic problems were found for either group (Hawes et al., 2015).

These differing results may be explained by several factors. First, Cheng and Mix (2014) delivered training in small groups (3–4 children) supervised by a researcher, while Hawes et al. (2015) administered classroom (group) training without direct supervision. Without the supervision of a researcher, reduced engagement with training may have contributed to the results of the Hawes et al. (2015) study. Second, post‐testing was delivered immediately following training by Cheng and Mix (2014), while Hawes et al. (2015) delivered post‐testing 1 week after training. Thus, caution must be taken in assuming that the gains reported by Cheng and Mix (2014) are durable. Third, the training method differed between the two studies. Implicit instruction was used by Hawes et al. (2015). Points were awarded for correct trials, but no instructions were given to explain correct (or incorrect) answers. In contrast, Cheng and Mix (2014) used explicit instruction, by giving participants physical manipulatives (mirroring those included in the onscreen trials) and instructing them to move the shapes to check their answers.

Differences in the training modes used in the above two studies reflect a broader distinction between explicit and implicit instruction types. In this study, implicit instruction is defined as instruction in which students are not aware of learning and use their experiences to construct an understanding. In contrast, for explicit instruction, the instructor plays a key role in explaining concepts to students and the student is aware of the skill or knowledge being taught. While there is mixed evidence regarding the effectiveness of explicit and implicit instruction in learning more generally (Kirschner, Sweller, & Clark, 2006), to our knowledge, no spatial training studies compare the efficacy of implicit and explicit instruction. Most studies of children have demonstrated the effectiveness of spatial training using implicit training, for example where participants complete task practice with feedback (Uttal et al., 2013). Instructional videos are one tool that can be used to deliver explicit instruction. There is evidence that viewing an instructional video of successful task completion can improve subsequent performance in number line estimation and spatial cross‐sectioning in adults (Cohen & Hegarty, 2014; Gallagher‐Mitchell, Simms, & Litchfield, 2018). The success of instructional videos may be attributable to observational learning (Castro‐Alonso, Ayres, & Paas, 2014; Paas & Sweller, 2012). In particular, for spatial thinking, instructional videos may activate the mirror neuron system as individuals imagine movements (Rizzolatti & Sinigaglia, 2010; Tettamanti et al., 2005). From a practical perspective, instructional videos could offer a novel, practical method of introducing spatial thinking into the classroom. To maximize the consistency of explicit instruction in this study, instructional videos were used. However, explicit instruction delivered by an individual, for example, a teacher or other expert, may have differing results and is not explored in this study.

Another factor that is not often considered in training studies, but that is controlled for in the current study, is the role of motivational factors. First, expectation (placebo) effects occur when the expectation that training will be effective induces cognitive gains, independently from the training content (Green et al., 2019). The placebo effect is well documented in medical domains with some limited evidence that expectation effects play a role in cognitive psychology studies (Dweck, 2000; Foroughi, Monfort, Paczynski, McKnight, & Greenwood, 2016; Jaeggi, Buschkuehl, Shah, & Jonides, 2014). By controlling for expectation effects, the causal inferences made in this cognitive training study are enhanced (Boot, Simons, Stothart, & Stutts, 2013). The degree to which participants engage with training is also proposed to impact training outcomes. For example, differences in participant engagement may explain the contrasting findings reported by Cheng and Mix (2014) and Hawes et al. (2015). In adult studies, those who show higher levels of engagement with cognitive training exhibit larger gains (Jaeggi et al., 2014). By controlling for participant engagement, the rigour of this study is substantially stronger, as it was possible to determine the extent to which cognitive training gains are attributable to training, over and above differences in participant engagement.

### 1.3 Current study

This study compared explicit and implicit instruction methods as means of training spatial skills in children aged 8 years and explored transfer of spatial training gains to other spatial and mathematics domains. Explicit instruction was delivered using instructional videos which were designed for use in this study. The choice of spatial scaling and mental rotation as spatial training targets was supported by both theoretical and behavioural evidence. The effectiveness of the intervention was assessed in the context of near, intermediate and far transfer of gains. A further original aspect of this study is that motivational factors including engagement with, and expectations of spatial training were controlled for.

## 2 METHODS

### 2.1 Participants

The sample size for this study was determined using GPower. The power analysis was based on the largest analysis completed in this study (3 × 2 × 2 ANOVA). To achieve power of 0.8, with a medium effect size (*f* = 0.25), power analysis indicated that a minimum of 158 participants were required. As the study design included data collection at two‐time points, it was anticipated that there would be some participant drop‐off between Time 1 and Time 2. Therefore, the sample size was increased to account for possible attrition of the sample. Participants were 250 children from six primary schools across London, UK. All participants were in Year 3 (*M*_age_ = 8.09 years, *SD* = 0.41 years). The overall proportion of males (48%) and females (52%) was approximately equal. Participant demographics across training groups are shown in Table 1.

**Table 1:** Demographic information across training groups

Training type	Training mode	*N*	Gender (% female)	Age (mean ± *SD*)
Mental rotation	Explicit	44	45.5	8.011 ± 0.438
	Implicit	42	59.5	8.052 ± 0.306
Spatial scaling	Explicit	41	51.2	8.151 ± 0.321
	Implicit	43	48.8	8.047 ± 0.474
Control	Explicit	41	53.7	7.942 ± 0.446
	Implicit	39	51.3	8.344 ± 0.291

### 2.2 Study design

**Figure 1:**
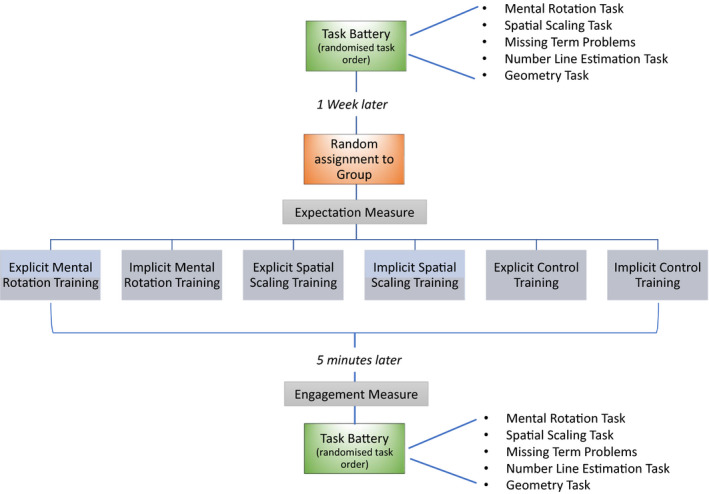
Overview of the study design

As shown in Figure 1, this study used a randomized, controlled, pre‐post training design. All participants completed an identical battery of tasks 1‐week pre‐training ± 1 day (Time 1), and immediately (within 5 min) post‐training (Time 2). All tasks and training procedures were computer‐based and were delivered using Gorilla software (www.gorilla.sc). Participants completed testing in their school IT suites in groups of 6–8 participants supervised by at least one (but typically two) researchers. All task instructions were incorporated into the Gorilla software and were presented to participants using earphones. Participants moved through the task battery at their own pace. Data collection was completed over a 7‐month period (April–October).

### 2.3 Training procedures

Training groups differed by training mode (explicit vs. implicit) and training type (mental rotation vs. spatial scaling vs. control). For both implicit and explicit instruction, training lasted between 3 and 4 min. For implicit instruction, the length of training was dependent on participants' performance (i.e. the speed taken to complete the items). For some participants in the implicit instruction group, training lasted up to 6 min.

This combination of two possible training modes and three possible training types led to six groups. Participants were randomly assigned to a group immediately preceding training (see Table 1). Allocation was completed using the balanced randomization function on the Gorilla software. The total number of predicted participants was entered into the software before data collection (*N* = 240). As this study has six training groups, a ratio of 40:40:40:40:40:40 participants in each group was assumed. Assignment using balanced randomization in Gorilla is like a weighted dice roll. This means that the first participant to complete the study had a 40/240 chance of being assigned to each group. However, if for example participant 1 was assigned to explicit mental rotation training, the second participant would have a 39/240 chance of being assigned to explicit mental rotation training and a 40/240 chance of being assigned to each of the other training groups. This randomization ensures approximately equal numbers of participants in each group. Any differences in group sizes are attributable to (a) data loss following group assignment; (b) additional participants being included in the study. Any additional participants (beyond the predicted 240) were assigned to groups using unbalanced randomization, that is, the probability that they were assigned to each group was 1/6 and was not dependent on the assignment of prior participants.

#### 2.3.1 Explicit training

**Figure 2:**
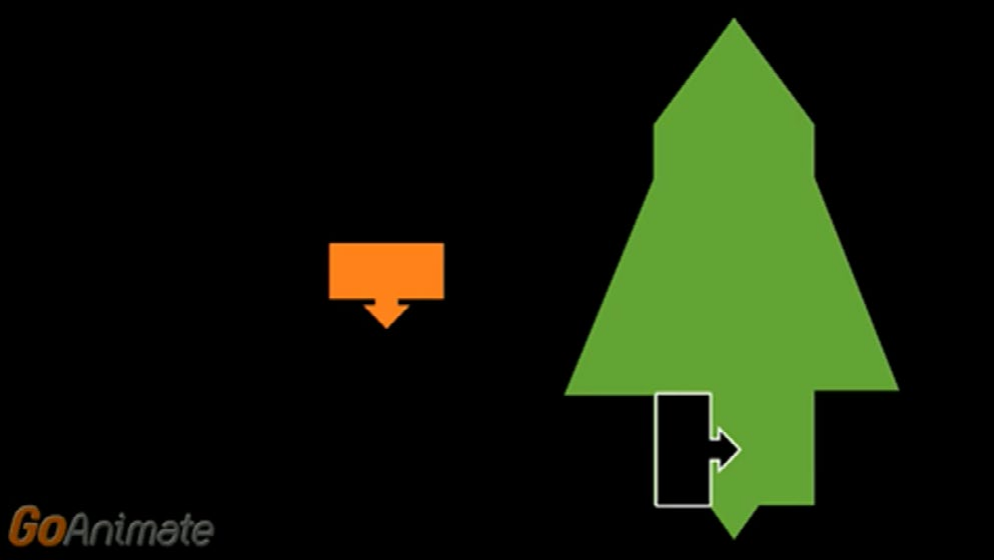
Screenshot taken from the instructional video of mental rotation (explicit instruction)

**Figure 3:**
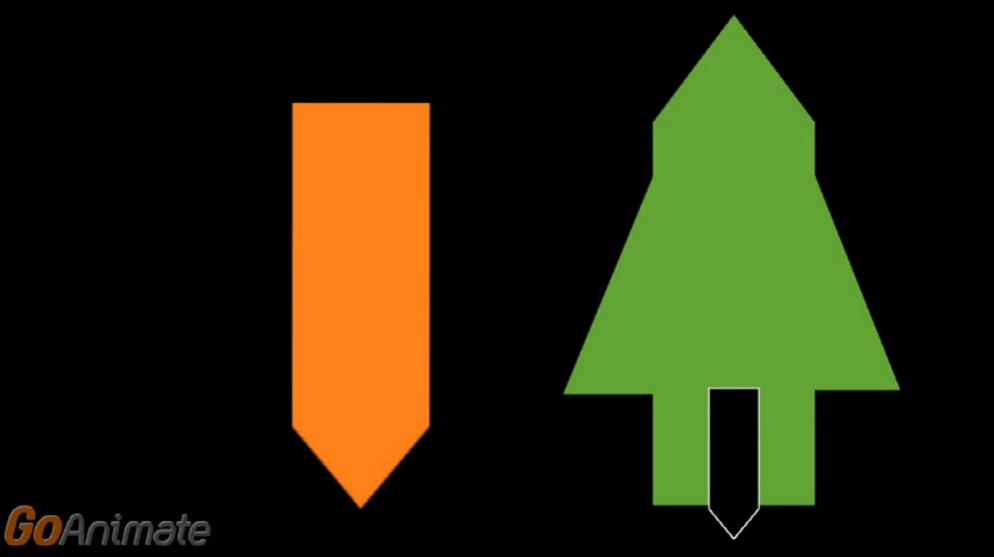
Screenshot taken from the instructional video of spatial scaling (explicit instruction)

**Figure 4:**
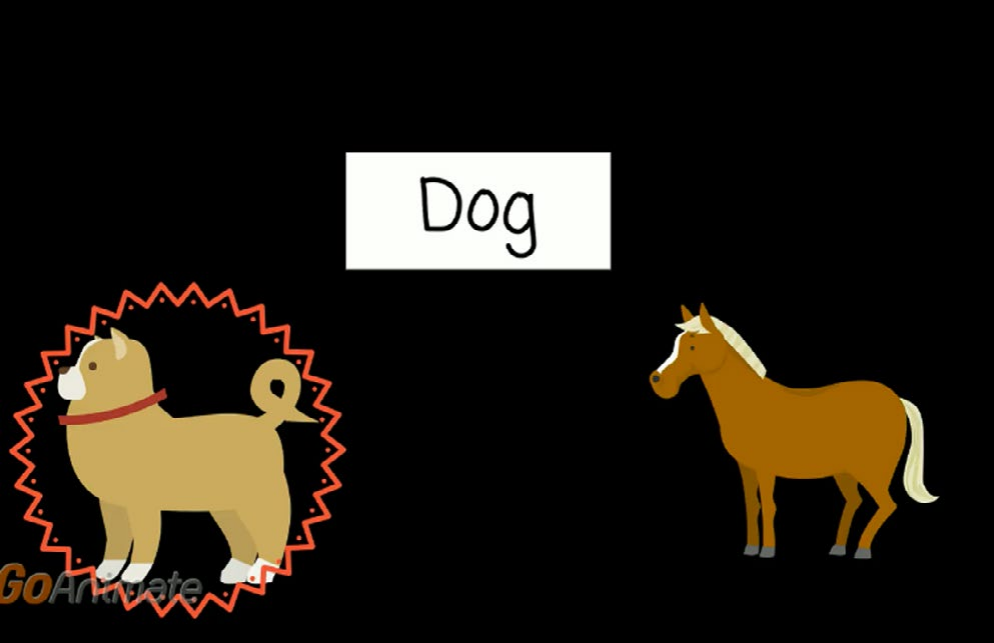
Screenshot taken from the control instructional video (explicit instruction)

Three of the training groups viewed instructional videos that provided explicit task instructions. Two groups watched videos with spatial content, while the control group watched a video on word reading. The videos were designed using Vyond (www.vyond.com). All non‐training content was uniform across videos, for example, the characters, storyline and narration. The videos can be accessed using the links provided below. Group 1 viewed the instructional mental rotation video. Participants in this group were given a description and viewed eight examples of mental rotation (see Figure 2 for a screenshot). For more details go to https://youtu.be/18iyRsvtGAQ. Group 2 viewed the instructional scaling video, in which a description of spatial scaling, and eight examples of spatial scaling were shown (see Figure 3). For more details go to https://youtu.be/grhxFEqgz51. For Group 3, the control video was shown. Participants watched eight examples of word‐picture matching, in which the onscreen characters selected the correct picture to match a given word (see Figure 4). Participants allocated to this control group did not view any spatial‐related content. For more details go to https://youtu.be/qDmgRR2RLyE.

#### 2.3.2 Implicit training

**Figure 5:**
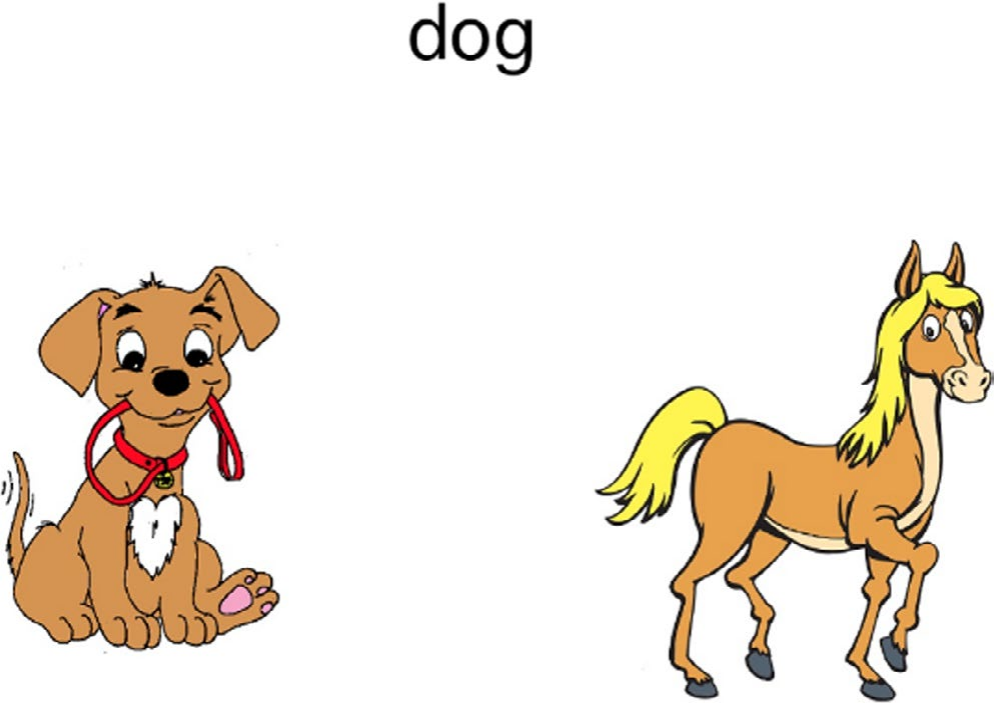
Sample trial from control training (implicit instruction)

The three implicit training groups completed task practice with computer‐based feedback. For each trial, participants were shown an onscreen tick or cross indicating the accuracy of their response. For incorrect trials, participants were given the opportunity to repeat the trial until they had selected the correct answer (all tasks had two possible response options). Participants were not given any explicit instruction on how to complete the trials. Participants moved to the next trial when the correct response was selected. For implicit training, two groups completed spatial tasks (the same tasks presented at Time 1), while a control group completed a word reading task. The number of trials included in implicit training was determined as the approximate number of trials that could be completed in the same length of time as the explicit instruction. This was established through piloting. Group 4 completed implicit mental rotation training and were presented with 30 trials of the Mental Rotation Task with feedback (further details of this task are outlined below). Group 5 completed implicit scaling training comprising of 24 trials of the Spatial Scaling Task (further details of this task can be found below). Feedback was given for each trial. Group 6 completed implicit control training. These participants completed 30 trials of a Word‐Picture Matching Task in which they were asked to match a word to one of two pictures using labelled keys on the keyboard (see Figure 5). This was a reading task requiring minimal spatial skills. Feedback was provided.

### 2.4 Task battery

The task battery included two spatial measures, assessing mental rotation and spatial scaling respectively. These measures were included as potential targets of near transfer (spatial tasks trained on) and of intermediate transfer (untrained spatial tasks). Three mathematics measures were included in the task battery as potential targets for far transfer (missing term problems, a Number Line Estimation Task and a Geometry Task). The order of task presentation was randomized across participants at both time points. To assess the role of motivational factors, two participant engagement measures were also administered.

#### 2.4.1 Mental Rotation Task

**Figure 6:**
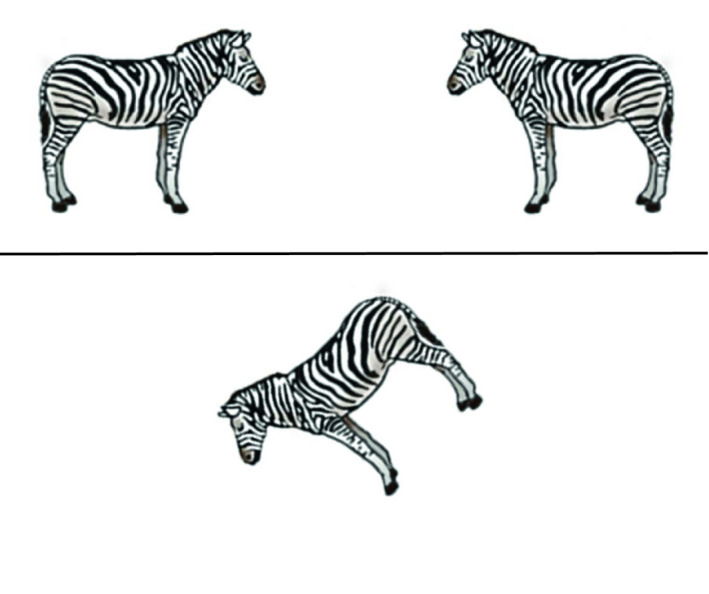
Sample stimulus from the Mental Rotation Task (45° anti‐clockwise trial)

In each trial of the Mental Rotation Task participants were required to identify which of two animal images located above a horizontal line matched the target image below the line. As shown in Figure 6, the images above the line included a mirror image of the target image, and a version of the target image rotated by a fixed degree from the target image. Participants used labelled keys on the computer keyboard to respond. Trials were separated by a fixation dot displayed for 500 ms. The task stimuli were taken from Neuburger, Jansen, Heil, and Quaiser‐Pohl (2011). Participants completed four practice trials at 0° where feedback was provided. Only participants achieving at least 50% in the practice trials continued to the 40 experimental trials. No feedback was given for experimental trials at Time 1 or Time 2. The experimental trials included equal numbers of clockwise and anti‐clockwise rotations at 45°, 90° and 135° (eight trials for each degree of rotation), and eight trials at 180° and 0°. The order of trial presentation was randomized for each participant. Percentage accuracy was recorded.

#### 2.4.2 Spatial Scaling Task

**Figure 7:**
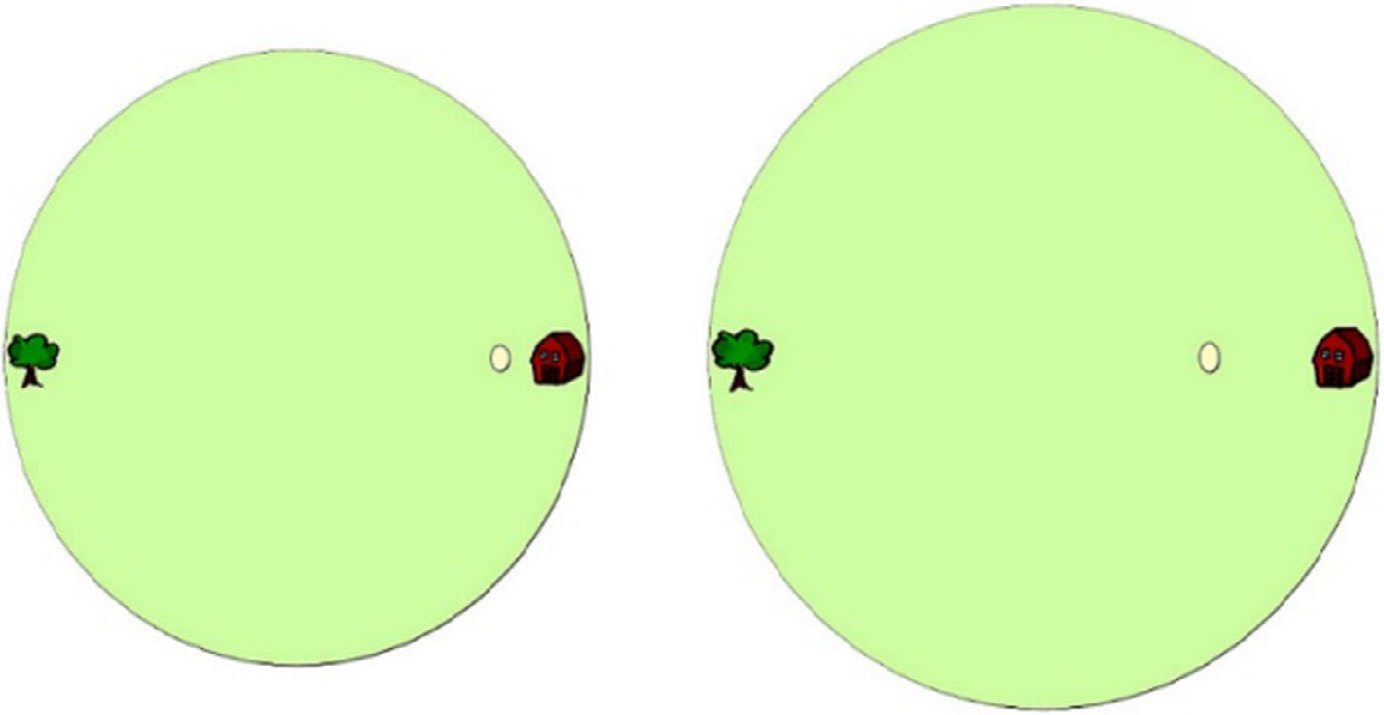
Sample mismatch trial at a scaling factor of 0.875 from the Spatial Scaling Task (taken from Möhring et al., 2016)

The Spatial Scaling Task was modified from Möhring, Newcombe, and Frick (2016). In each trial participants were shown two 2D images of a circular space (a farmer's field) containing a target (an egg). Participants were asked to identify whether the eggs in the two fields were in the same position or in different positions (see Figure 7). For half of the trials, the targets were presented in the same position in both fields (match trials). For the remaining trials, the position of the target in one field was adjusted by 2 cm (to the left or right) relative to the second field (mismatch trials). Participants responded using labelled keys on the computer keyboard. All trials were separated by a fixation dot displayed for 500 ms. Participants completed six practice trials during which feedback was given and no time limit was imposed. Only participants achieving at least 50% in the practice trials continued to the experimental trials. The 72 randomly experimental trials were presented randomly. Each trial was displayed for 5 s. No feedback was given for experimental trials at Time 1 or Time 2. Experimental trials differed by the location of the target on the horizontal axis, and by scaling factor. Six different target positions were included (a modification from the original study where 15 positions were used). Scaling factor was manipulated by keeping the size of one space constant while manipulating the size of the second. In this way six scaling factors were included (1, 0.875, 0.75, 0.625, 0.5, 0.375). Performance was measured as percentage accuracy.

#### 2.4.3 Missing term problems

**Figure 8:**
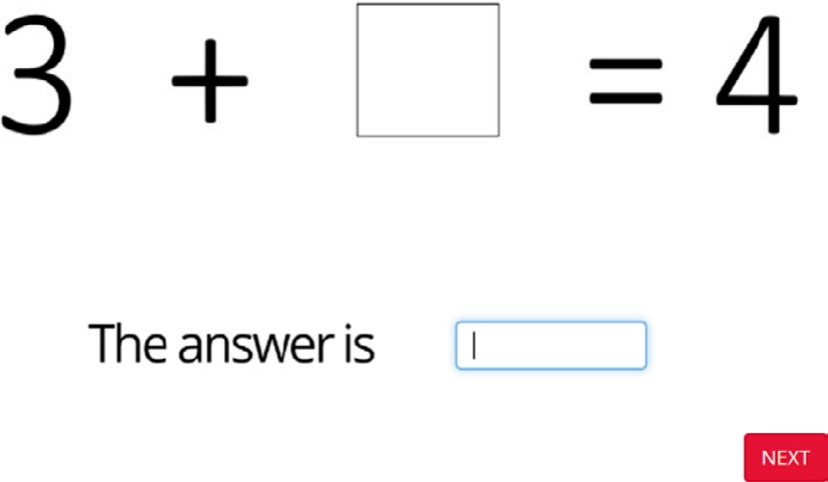
Sample missing term problem

The missing term problems included in this study were modified from Hawes et al. (2015). For each item participants were required to complete the missing number(s) in a simple mathematical equation (see Figure 8). This task included two practice items where the solutions were shown after participants submitted an answer. Following this, 21 test items were displayed. No solutions were shown for these items. Test items included the original 18 items from Hawes et al. (2015) and three additional, low‐difficulty items that were added to the task after piloting to alleviate floor effects. Items were presented in order of increasing difficulty and a time limit of 25 s was allocated to each test item. Approximately equal numbers of addition versus subtraction items, and single versus multi‐digit numbers were included. The position of the missing box was also balanced across items. Performance accuracy was recorded.

#### 2.4.4 Number Line Estimation Task

**Figure 9:**
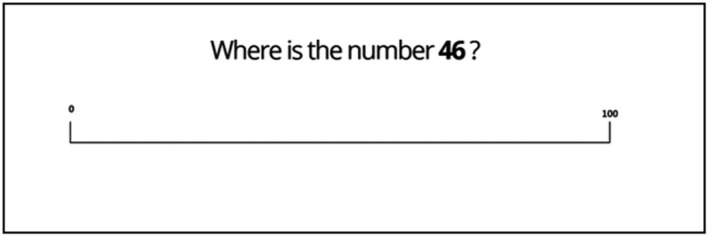
Sample item from the Number Line Estimation Task

The Number Line Estimation Task was used to measure numerical representations. The method was adapted from Siegler and Opfer (2003). As shown in Figure 9, for each item participants were presented with a target number and were asked to estimate its location on a 0–100 number line by using the mouse cursor to click the number line at their selected location. For practice items (*N* = 2) solutions were shown onscreen after participants attempted an answer. No solutions were given for experimental items (*N* = 30). The target numbers included in the task were taken from Gallagher‐Mitchell, Romero‐Rivas, Rodriguez‐Cuadrado, and Dackermann, (2017). The order of experimental items was randomized. Performance was measured as percentage absolute error (PAE) and as logarithmic response patterns (*R* ^2^_LOG_; Simms, Clayton, Cragg, Gilmore, & Johnson, 2016). PAE is the numerical distance from a participant's answer to the correct answer, divided by the length of the number line. This measure reflects the accuracy of participants' estimates. For each participant, linear (*R* ^2^_LIN_) and logarithmic (*R* ^2^_LOG_) response patterns were also calculated using curve estimation. Curve estimation is based on the correlation between participants' estimates and the target numbers. The proximity of *R* ^2^_LIN_ and *R* ^2^_LOG_ scores to the value of 1 is an indicator of how well a participant's estimates reflect a linear or logarithmic pattern respectively.

#### 2.4.5 Geometry Task

**Figure 10:**
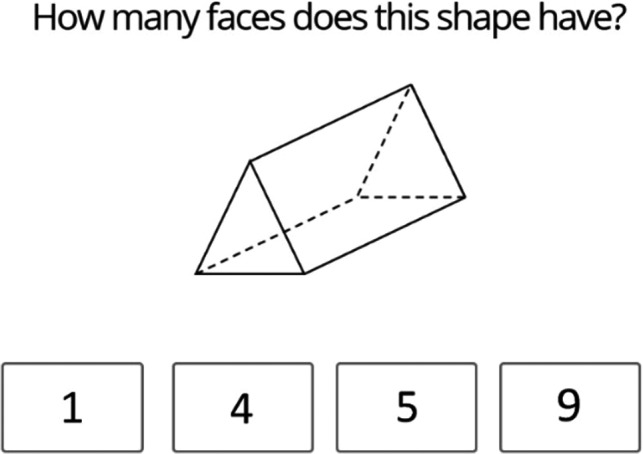
Sample 3‐D shape item from the Geometry Task

The Geometry Task was designed for this study based on the statutory geometry learning requirements for Year 2 students in the UK (Department of Education, 2013). The task included two item types, Shape Items and Symmetry Items. For Geometry Shape Items, participants were shown an image of a shape and were asked to select the correct number of sides (or faces) on the shape from four possible response options (see Figure 10). Participants completed a single practice item using a 2‐D shape on which they were given feedback. All participants successfully completed this item. Geometry Shape Items differed in the dimensionality of the images shown and included six 2‐D shapes and six 3‐D shapes. Performance was measured as accuracy across all items.

**Figure 11:**
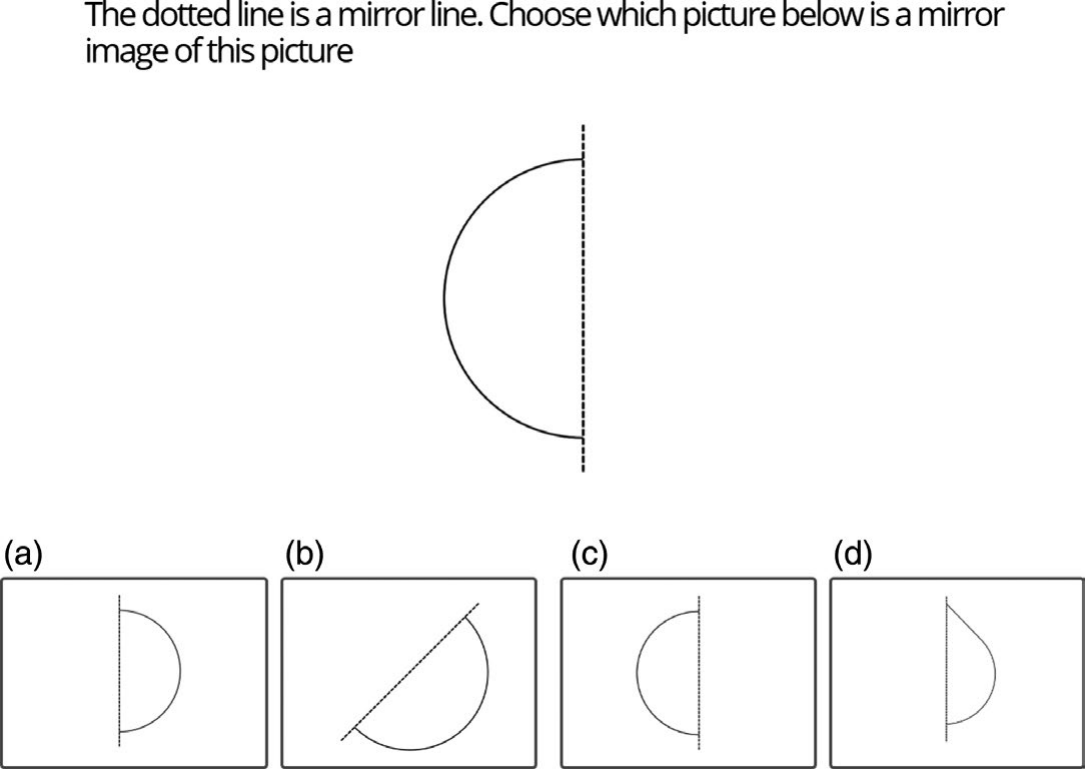
Sample Geometry Symmetry Item showing a match error (a), a shape error (b), a symmetry error (c) and the correct answer (d)

For each Geometry Symmetry Item, a target shape was displayed on screen and participants were asked to select which of four possible response options was the mirror image of the target shape (see Figure 11). Participants completed a single practice trial in which they received feedback. Ten experimental Symmetry Items were presented in a randomized order. For each item, the distractor images included a match error, a shape error and a symmetry error (see Figure 11). For match errors, the distractor was identical in both shape and position to the target shape (a). For shape errors, the distractor was in the correct position, however the shape was not a mirror of the target image, but another similar shape (b). Finally, for symmetry errors the distractor was the correct shape however the position of the distractor was not an accurate mirror image (c). Performance accuracy was recorded.

#### 2.4.6 Expectations of the effectiveness of training

**Figure 12:**
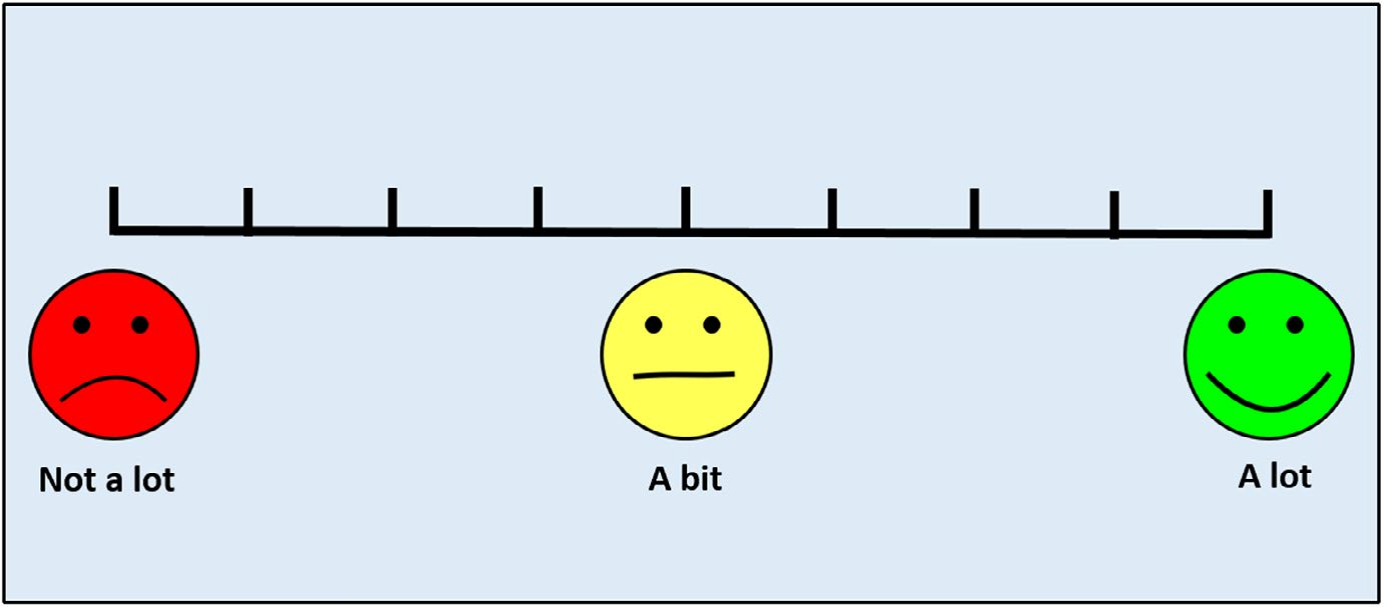
Response scale for measuring expectations of the effectiveness of training

Prior to the delivery of training, all participants were asked a single question, measuring their expectations of the effectiveness of training, ‘We are going to be playing some games. How much do you think the games will help you with your maths?’. The question was displayed alongside an onscreen scale (see Figure 12). Participants responded by selecting a point on the scale using the mouse cursor. Participant's responses were coded as 1–12 based on the onscreen position selected. A score of 1 was allocated for responses that indicated low expectations of training while a score of 12 was allocated for responses that indicated high expectations of training.

#### 2.4.7 Participant Engagement Questionnaire

**Figure 13:**
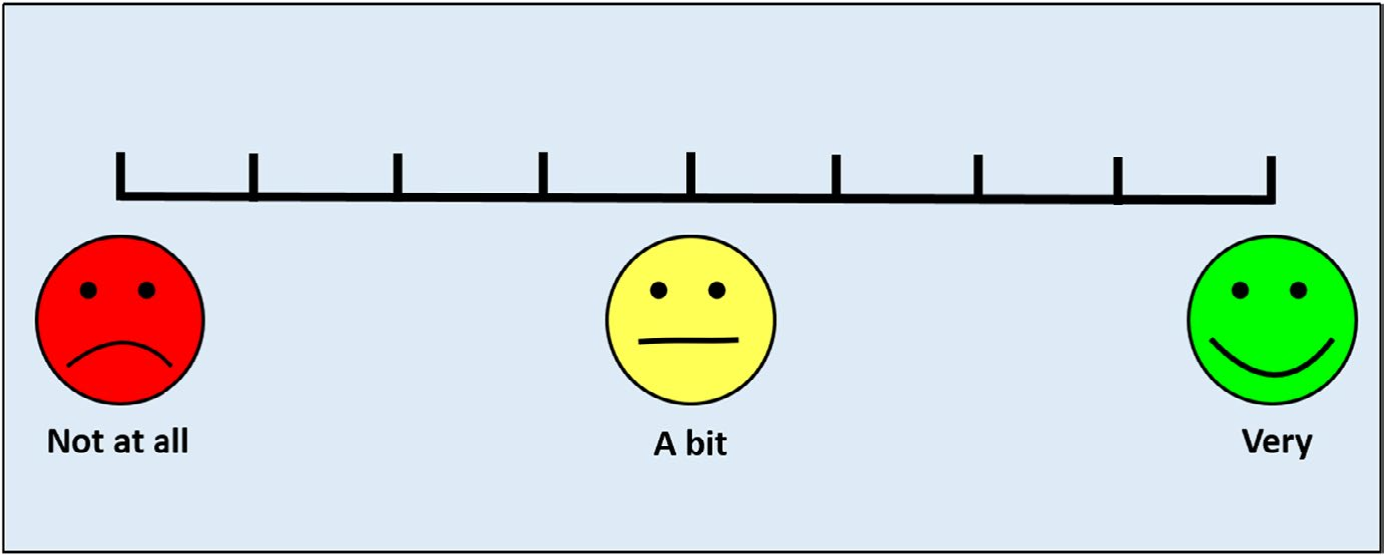
Sample scale from the Participant Engagement Questionnaire

A participant engagement questionnaire was delivered to assess participant's enjoyment of and engagement with the training that they had received. The questionnaire was designed for use in this study. As shown in Table 2, the questionnaire included four questions, the phrasing of which varied slightly based on the type of training delivered. Each question was presented alongside an onscreen scale (for an example see Figure 13). Participants responded to each question by selecting a point on the scale using the mouse cursor. Participant's responses were coded as 1–12 based on the onscreen position selected. A score of 1 was allocated for responses that indicated low engagement while a score of 12 was allocated for responses that indicated high engagement. Participants were awarded an overall engagement score, an average of their scores across all four questions (where necessary items were reverse coded).

**Table 2:** Items included in the Participant Engagement Questionnaire

Item	Explicit training	Implicit training
1	How much did you enjoy the video?	How much did you enjoy the game?
2	How exciting was the video?	How exciting was the game?
3	How easy was it to understand the video?	How easy was it to understand the game?
4	How much effort did it take to watch the video?	How much effort did it take to play the game?

### 2.5 Exclusion criteria

Due to technical errors and school disruptions, data for a single task was lost for nine participants at Time 1 and 15 participants at Time 2. These participants were excluded from training analysis for the task on which they were missing data. Furthermore, participants scoring higher than 95% on a given task at Time 1, were deemed to have reached “ceiling level” performance on the task and were excluded from training analysis for that task only. For missing term problems and Number Line Estimation, responses were open ended. For missing term problems, participants who did not score higher than 10% at Time 1, were not deemed to understand the task aims and were excluded (*n* = 14). For Number Line Estimation participants who didn't attempt at least 75% of items, or participants with a mean PAE score higher than 15% for practice items were also excluded (*n* = 0). Parametric analyses were used as all groups were large enough (*N* > 30) for the central limit theorem to apply (Field, 2013).

## 3 RESULTS

### 3.1 Performance at Time 1

#### 3.1.1 Overall performance at Time 1

No ceiling or floor effects were present for any measures (Table 3). Descriptive information for performance on each of the tasks, across groups is shown in Table 6. For the Geometry Task, the results of a dependent *t* test indicated a significant difference in performance between Geometry Shape Items (63.73 ± 1.05) and Geometry Symmetry Items (54.36 ± 2.08), *t*(1, 249) = 4.34, *p* < .001, *d* = 0.295. Furthermore, while a Pearson's correlation indicated a significant association between the different item types, *r*(248) = .178, *p* = .005, the correlation was small to medium in size, that is, between .1 and .3 (Field, 2013). Hence, Geometry Symmetry Items and Geometry Shape Items are considered separately throughout. For Number Line Estimation, 74% of participants had estimates that were best described by a linear (compared to a logarithmic) response pattern.

**Table 3:** Descriptive statistics at Time 1

Measure	Descriptive Statistics				
	Mean	*SE*	*SD*	Min	Max
Mental rotation	59.00	0.99	15.64	25.00	100.00
Spatial scaling	54.00	0.54	8.54	23.61	79.17
Missing box problems	56.42	1.56	24.68	0.00	100.00
Number line PAE	0.10	0.01	0.06	0.03	0.30
Number line *R* ^2^_LIN_	0.93	0.01	0.08	0.63	1.00
Geometry Shape Items	63.73	1.05	16.54	16.67	100.00
Geometry Symmetry Items	54.36	2.08	32.94	0	100.00
Expectations (mean rating 0–12)	9.47	0.23	3.64	0	12.00

Note

For this and all other analysis, unless otherwise stated all results reported are percentage correct scores.

#### 3.1.2 Gender differences in task performance at Time 1

Independent *T* tests (controlling for multiple comparisons [0.05/8 = 0.006]) were used to explore gender differences in performance at Time 1. Where homogeneity of variance could not be assumed, the results for unequal variances were reported. As shown in Table 4, males had significantly lower error scores on the Number Line Estimation Task compared to females, *t*(148) = 3.15, *p* = .002, *d* = 0.401. No other significant gender differences were reported (*p*s > .05, *d*s < 0.261). Thus, gender was included as a control variable when investigating the effects of training on the Number Line Estimation Task only.

**Table 4:** Gender differences in task performance at Time 1

Test measure	Gender				Statistics	
	Male (*n* = 121)		Female (*n* = 129)		Test statistic (*t*)	Effect size (*d*)
	Mean	*SD*	Mean	*SD*		
Mental rotation	60.382	16.053	57.761	15.194	0.742	0.094
Spatial scaling	54.764	7.533	53.284	9.359	1.372	0.174
Missing term problems	59.708	24.573	53.341	24.471	2.052	0.261
Number line estimation *R* ^2^_LIN_	0.093	0.051	0.115	0.062	1.435	0.182
Number line estimation PAE	0.938	0.073	0.924	0.084	3.154*, ***	0.401
Geometry Shape Items	62.810	15.592	64.596	17.390	0.853	0.108
Geometry Symmetry Items	53.554	33.834	55.116	32.189	0.374	0.047
Expectations	9.126	3.864	9.791	3.390	1.449	0.184

* *p* < .05.

#### 3.1.3 Differences in task performance across training groups at Time 1

To confirm that there were no performance differences between groups at Time 1, a two‐way ANOVA was completed for each task. Training mode (2 levels: explicit vs. implicit) and training type (3 levels: mental rotation vs. spatial scaling vs. reading) were included as between participant variables. Comparing across training types and training modes, no significant differences in performance were reported for any of the mathematics or spatial tasks (*p *> .05, $\eta_{p}^{2}$ < 0.010; see Table 6). Similarly, there were no differences in expectations of training across training modes, *F*(1, 244) = 3.25, *p* = .072, $\eta_{p}^{2}$ = 0.013, or training types, *F*(2, 244) = 0.27, *p* = .763, $\eta_{p}^{2}$ = 0.002.

#### 3.1.4 Associations between measures at Time 1

Pearson correlations were completed between measures at Time 1. This allowed for the investigation of whether the observed associations between spatial and mathematics skills that have been demonstrated in previous studies (e.g. Gilligan et al., 2018; Mix et al., 2016) and form the rationale for the training paradigm used in this study, were present. As shown in Table 5, significant correlations were reported between all tasks, except for performance on Geometry Shape Items which was not correlated with mental rotation accuracy, *r*(248) = 0.09, *p* = .147. Expectations of the effectiveness of training were not correlated with performance on any behavioural measures.

**Table 5:** Bivariate correlations between tasks at Time 1

	Spatial tasks		Mathematics tasks					Expectations
	1	2	3	4	5	6	7	8
1. Mental rotation	**—**	0.275*, ***	0.293*, ***	−0.213*, ***	0.247*, ***	0.092	0.227*, ***	0.057
2. Spatial scaling		**—**	0.345*, ***	−0.304*, ***	0.333*, ***	0.160*	0.258*, ***	0.037
3. Missing box problems			**—**	−0.492*, ***	0.531*, ***	0.303*, ***	0.421*, ***	−0.021
4. Number line PAE				**—**	−0.825*, ***	−0.254*, ***	−0.327*, ***	0.014
5. Number line *R* ^2^_LIN_					**—**	0.223*, ***	0.305*, ***	−0.023
6. Geometry Shape Items						**—**	0.178*, ***	0.013
7. Geometry Symmetry Items							**—**	−0.032
8. Expectations								**—**

* *p* < .05

*** *p* < .001.

### 3.2 Performance at Time 2

Mixed ANOVAs were used to investigate training effects across near, intermediate, and far transfer measures (see Table 6 for a summary of performance scores across Time 1 and Time 2). Time was included as a within participant variable (Time 1 and Time 2). Training mode (explicit vs. implicit) and training type (mental rotation vs. spatial scaling vs. control) were included as between participant variables. Where sphericity could not be assumed, Greenhouse‐Geisser values were reported. It is noteworthy that ANCOVAs with Time 2 scores as the dependent variable and Time 1 scores as a covariate were run in parallel to these analyses. Comparable results were reported for all outcomes. Further details, including comparisons between training types at Time 2, can be found in the Supporting Information.

**Table 6:** Performance at Time 1 and Time 2 across groups

Training type	Mode	Mental rotation percent correct		Spatial scaling percent correct		Missing term problems percent correct	
		Time 1	Time 2	Time 1	Time 2	Time 1	Time 2
Mental rotation	Explicit	56.904 ± 15.287	65.262 ± 17.916	54.040 ± 8.153	55.240 ± 9.270	59.181 ± 22.152	66.795 ± 22.796
	Implicit	54.421 ± 12.616	64.634 ± 17.559	52.966 ± 7.045	51.464 ± 8.630	52.092 ± 21.127	55.411 ± 25.333
Spatial scaling	Explicit	58.232 ± 15.590	64.141 ± 18.448	55.015 ± 8.348	59.568 ± 9.424	59.867 ± 19.854	58.687 ± 24.617
	Implicit	61.698 ± 14.775	61.539 ± 17.607	55.386 ± 8.921	59.079 ± 9.802	59.774 ± 19.764	55.890 ± 23.029
Control	Explicit	60.703 ± 15.474	60.391 ± 17.964	54.268 ± 9.309	53.286 ± 8.832	58.377 ± 24.473	58.201 ± 24.916
	Implicit	60.657 ± 16.748	61.619 ± 16.284	51.816 ± 9.702	51.175 ± 9.320	60.784 ± 19.336	57.843 ± 22.554

Training type	Mode	Number line *R* ^2^_LIN_		Number line PAE		Geometry Shape Items accuracy	
		Time 1	Time 2	Time 1	Time 2	Time 1	Time 2
Mental rotation	Explicit	0.939 ± 0.080	0.917 ± 0.102	0.092 ± 0.051	0.111 ± 0.069	61.585 ± 15.797	62.602 ± 20.927
	Implicit	0.925 ± 0.080	0.908 ± 0.088	0.108 ± 0.059	0.116 ± 0.066	62.281 ± 15.468	71.272 ± 18.247
Spatial scaling	Explicit	0.917 ± 0.098	0.923 ± 0.082	0.115 ± 0.065	0.106 ± 0.059	59.553 ± 15.316	64.431 ± 15.593
	Implicit	0.941 ± 0.073	0.936 ± 0.064	0.104 ± 0.065	0.092 ± 0.048	67.906 ± 13.923	73.246 ± 17.238
Control	Explicit	0.923 ± 0.075	0.891 ± 0.126	0.112 ± 0.062	0.152 ± 0.095	61.816 ± 15.940	58.761 ± 19.113
	Implicit	0.937 ± 0.072	0.896 ± 0.124	0.105 ± 0.056	0.119 ± 0.065	59.460 ± 13.489	61.711 ± 14.361

Training type	Mode	Geometry Symmetry Items accuracy	
		Time 1	Time 2
Mental rotation	Explicit	52.683 ± 31.861	64.878 ± 28.118
	Implicit	48.684 ± 27.428	62.895 ± 30.395
Spatial scaling	Explicit	43.156 ± 32.292	62.632 ± 34.772
	Implicit	53.889 ± 30.917	63.889 ± 29.498
Control	Explicit	43.514 ± 34.818	54.324 ± 35.162
	Implicit	63.333 ± 28.983	68.889 ± 26.702

Abbreviations: PAE, percentage absolute error, *R* ^2^_LIN_, linear response pattern.

#### 3.2.1 Near and intermediate transfer of gains

##### Mental rotation

**Figure 14:**
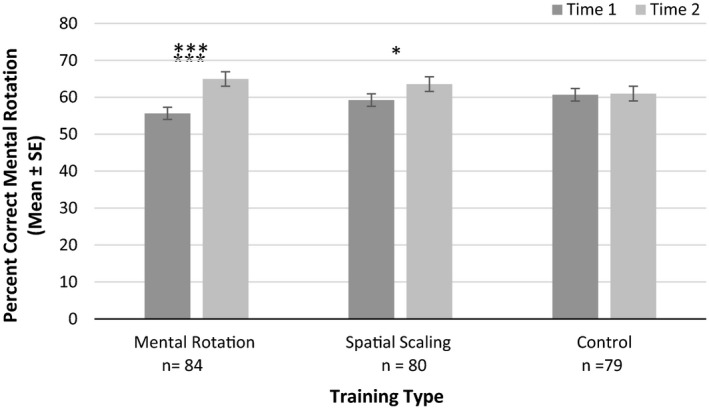
Mental rotation accuracy at Time 1 and Time 2 for different training types (*p < .05, **p < .01, ***p < .001)

A significant main effect of time was reported, with higher performance at Time 2, *F*(1, 237) = 21.87, *p* < .001, $\eta_{p}^{2}$ = 0.084. A significant interaction was found between time and training type, *F*(2, 237) = 6.88, *p* < .001, $\eta_{p}^{2}$ = 0.055. As shown in Figure 14, paired sample *t* tests indicated a significant improvement in performance accuracy following mental rotation training, *t*(83) = 5.49, *p* < .001, *d* = 0.581 (near transfer) and spatial scaling training, *t*(79) = 2.30, *p* = .024, *d* = 0.263 (intermediate transfer). No significant improvement in performance accuracy was reported following control training, *t*(78) = 0.21, *p* = .837, *d* = 0.019. No other main effects or interactions with time were reported (*p*s > .05, $\eta_{p}^{2}s$ < 0.005).

##### Spatial scaling

**Figure 15:**
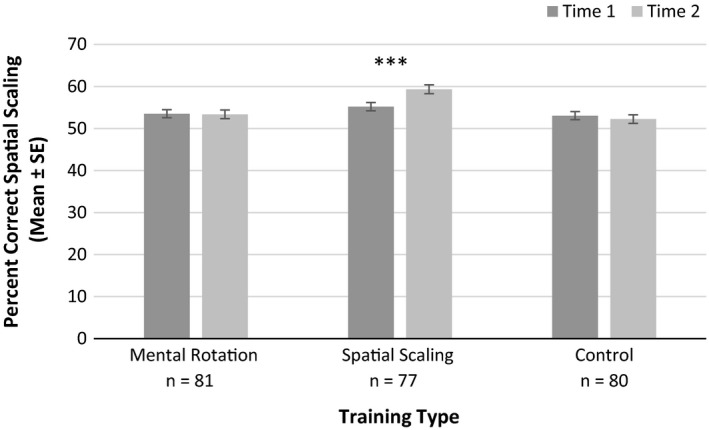
Spatial scaling accuracy at Time 1 and Time 2 for different training types (*p < .05, **p < .01, ***p < .001)

A significant main effect of training type was found, with higher performance for spatial scaling training compared to the other training types, *F*(2, 232) = 8.28, *p* < .001, $\eta_{p}^{2}$ = 0.067. There was also a significant interaction reported between time and training type, *F*(2, 232) = 6.25, *p* = .002, $\eta_{p}^{2}$ = 0.051 (see Figure 15). Paired sample *t* tests indicated significant performance gains following spatial scaling training only, *t*(76) = 3.99, *p* < .001, *d* = 0.450 (near transfer). No significant gains were reported following mental rotation training, *t*(80) = 0.04, *p* = .972, *d* = 0.004, or control training, *t*(79) = 0.70, *p* = .485, *d* = 0.088. There were no other main effects or significant interactions with time (*p*s > .05, $\eta_{p}^{2}s$ **< 0.005).

#### 3.2.2 Far transfer of gains

##### Missing term problems

**Figure 16:**
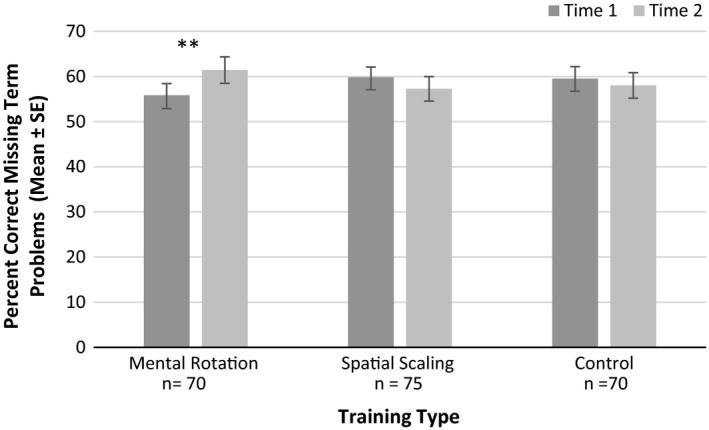
Percentage correct on missing term problems at Time 1 and Time 2 for different training types (*p < .05, **p < .01, ***p < .001)

A significant interaction between time and training type was found, *F*(2, 209) = 4.58, *p* = .011, $\eta_{p}^{2}$ = 0.042 (see Figure 16). Paired sample *t* tests indicated a significant improvement in accuracy following mental rotation training only, *t*(69) = 2.73, *p* = .008, *d* = 0.241 (far transfer). No significant improvements were reported following spatial scaling training, *t*(74) = 1.30, *p* = .197, *d* = 0.117, or control training, *t*(69) = 0.73, *p* = .466, *d* = 0.067. There were no other significant main effects or interactions with time (*p*s > .05, $\eta_{p}^{2}s$ **< 0.009).

##### Number Line Estimation

**Figure 17:**
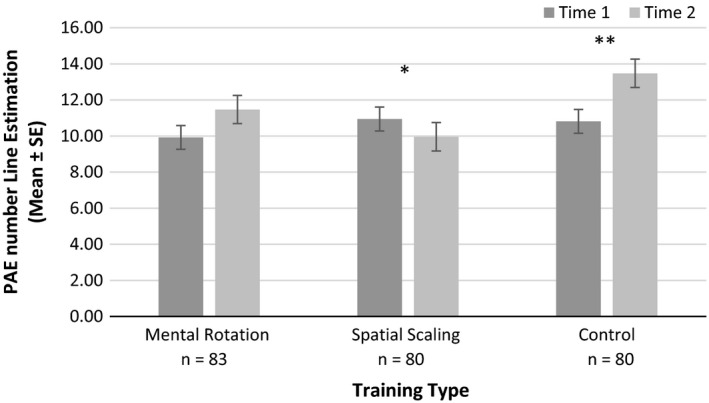
Percentage absolute error (PAE) on the Number Line Estimation Task at Time 1 and Time 2 for different training types (*p < .05, **p < .01, ***p < .001)

As a significant gender effect was reported for PAE scores on this task at Time 1, gender was included as a between participant variable. However, no significant main effect or interactions with gender were reported for this task (*p*s > .05, $\eta_{p}^{2}s$ **< 0.014). Hence, gender was removed, and the analysis was repeated. A significant main effect of time was reported, *F*(1, 237) = 5.86, *p* = .016, $\eta_{p}^{2}$ = 0.024. There was also a significant interaction between time and training type. As shown in Figure 17, there was a significant interaction between time and training type for PAE scores, *F*(2, 237) = 6.05, *p* = .002, $\eta_{p}^{2}$ = 0.054. Paired sample *t* tests indicated a significant reduction in error following spatial scaling training, *t*(79) = 2.12, *p* = .037, *d* = 0.236 (far transfer). No significant difference in error was found following mental rotation training, *t*(82) = 1.91, *p* = .060, *d* = 0.209. However, a significant increase in error was reported following control training, *t*(79) = 3.01, *p* = .003, *d* = 0.330. No other main effects or significant interactions with time were reported (*p*s > .05, $\eta_{p}^{2}s$ < 0.005). Similar analysis was completed for *R* ^2^_LIN_ performance. The patterns of performance across time and training type were comparable to PAE scores. Further information is available on request.

##### Geometry performance

**Figure 18:**
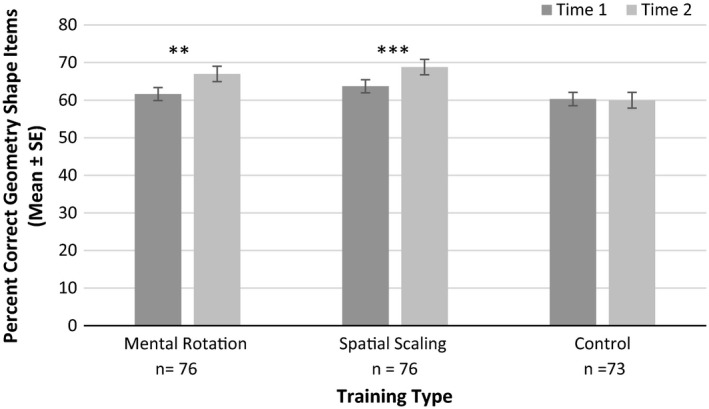
Accuracy on Geometry Shape Items at Time 1 and Time 2 for different training types (*p < .05, **p < .01, ***p < .001)

**Figure 19:**
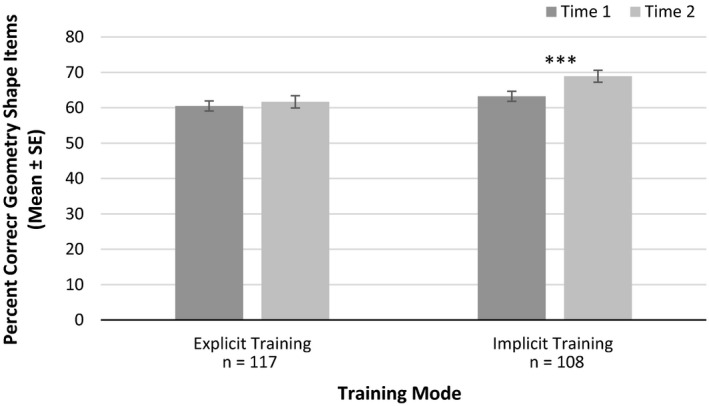
Accuracy on Geometry Shape Items at Time 1 and Time 2 for different training modes (*p < .05, **p < .01, ***p < .001)

For Geometry Shape Items there were main effects of time, *F*(1, 219) = 12.93, *p* < .001, $\eta_{p}^{2}$ = 0.056, training mode, *F*(1, 219) = 6.39, *p* = .012, $\eta_{p}^{2}$ = 0.028, and training type, *F*(2, 219) = 3.25, *p* = .041, $\eta_{p}^{2}$ = 0.029. There was also a significant interaction between time and training type for Geometry Shape Items, *F*(2, 219) = 3.82, *p* = .022, $\eta_{p}^{2}$ = 0.034 (see Figure 18). Paired sample *t* tests, indicated significant gains in performance accuracy following mental rotation training, *t*(75) = 2.93, *p* = .004, *d* = 0.308 (far transfer), and spatial scaling training, *t*(75) = 3.70 *p* < .001, *d* = 0.314 (far transfer). There were no significant gains following control training, *t*(72) = 0.21, *p* = .833, *d* = 0.024. There was also a significant interaction between time and training mode for Geometry Shape Items, *F*(1, 219) = 5.95, *p* = .016, $\eta_{p}^{2}$ = 0.026 (see Figure 19). There was a significant improvement in performance following implicit training, *t*(104) = 4.41, *p* < .001, *d* = 0.351, but not explicit training, *t*(116) = 0.85, *p* = .395, *d* = 0.069. No significant three‐way interaction between time, training mode and training type was reported *F*(2, 219) = 1.60, *p* = .204, $\eta_{p}^{2}$ = 0.014. For Geometry Symmetry Items, all groups had improved performance between Time 1 and Time 2, *F*(1, 213) = 40.30, *p* < .001, $\eta_{p}^{2}$ = 0.159. However, there were no other main effects or significant interactions with time (*p*s > .05, $\eta_{p}^{2}s$ **< 0.013).

#### 3.2.3 Motivational factors

##### Expectations of training

An ANOVA was completed with training mode and training type as between participant variables and expectations of training as the dependent variable. There were no significant differences in self‐reported expectations of training across training modes, *F*(1, 244) = 3.25, *p* = .072, $\eta_{p}^{2}$ *=* 0.013, or training types, *F*(2, 244) = 0.27, *p* = .763, $\eta_{p}^{2}$ = 0.002. ANCOVAs were also used to explore whether individual participant gains on each outcome measure were predicted by expectations of training. A separate ANCOVA was completed for each training type group (mental rotation, spatial scaling and control) and each training mode group (explicit and implicit). Time was included as a between participant variable and expectation score was included as a covariate. There were no significant interactions between participant expectations of training and time for any of the training types (*p*s > .05, $\eta_{p}^{2}s$ **< 0.033) or any of the training modes (*p*s > .05, $\eta_{p}^{2}s$ **< 0.012).

##### Participant engagement with training

**Figure 20:**
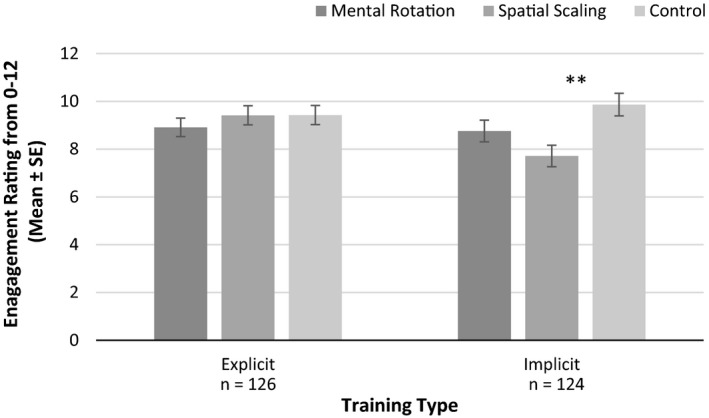
Self‐reported levels of engagement following training, across training modes and training types (*p < .05, **p < .01, ***p < .001)

An ANOVA was completed with training type and training mode as between participant variables and self‐reported engagement levels as the dependent variable. There was a significant difference in engagement across training types, *F*(2, 244) = 3.37, *p* = .036, $\eta_{p}^{2}$ = 0.027. Bonferroni pairwise comparisons indicated significantly higher engagement levels following control training compared to spatial scaling training (*p* = .034). There was no main effect of training mode on engagement, *F*(1, 244) = 1.81, *p* = .180, $\eta_{p}^{2}$ = 0.007. However, there was a significant interaction between training type and training mode on engagement, *F*(2, 244) = 3.30, *p* = .039, $\eta_{p}^{2}$ = 0.026. For explicit training there were no differences in engagement across training types, *F*(2, 123) = 0.56, *p* = .573, $\eta_{p}^{2}$ = 0.009. For implicit training there was an effect of training type, *F*(2, 121) = 5.42, *p* = .006, $\eta_{p}^{2}$ = 0.082. As highlighted in Figure 20, post‐hoc Bonferroni tests indicated significantly higher engagement following control training compared to spatial scaling training (*p* = .004).

## 4 DISCUSSION

The results reported support and extend previous correlational findings on spatial‐mathematical relations and provide insight into the causal relationships between different aspects of spatial and mathematical thinking. It was demonstrated that training mental rotation and, for the first time, training spatial scaling, led to gains in spatial and mathematical thinking at 8 years. These gains were present following explicit and implicit instruction. Spatial training gains had near, intermediate and far transfer effects. Spatial thinking is therefore one cognitive domain in which transfer of cognitive training gains is possible. The gains reported reflect the importance of choosing developmentally sensitive, theoretically motivated training targets.

*Near transfer*: Mental rotation and spatial scaling training led to significant gains in mental rotation, and spatial scaling respectively. Findings which are consistent with previous evidence that spatial skills are malleable in children (Uttal et al., 2013). Previous studies typically investigated the malleability of mental rotation or other spatial tasks that elicit mental visualization (Uttal et al., 2013) while this is the first study to highlight the malleability of spatial scaling in children at 8 years.

*Intermediate transfer*: Significant gains in mental rotation were reported following spatial scaling training providing evidence of intermediate transfer of spatial scaling training to an untrained spatial task. These findings are consistent with those of Uttal et al. (2013) who found that spatial training transferred to other untrained spatial tasks. However, Uttal et al. (2013) reported that intermediate transfer was not evident in all studies and was more likely to occur where longer training sessions were included. The short training sessions used in this study (3–5 min) may explain why no intermediate transfer was reported following mental rotation training.

*Far transfer*: Participants who completed mental rotation training had significant accuracy gains on missing term problems. The findings of far transfer of gains are consistent with the findings of Cheng and Mix (2014) who demonstrated that explicit mental rotation training led to gains in performance accuracy on a similar missing box task. Cheng and Mix (2014), proposed that these findings are due to the fact that children solve arithmetic problems of this type by mentally rotating the terms, thus restructuring the equation in a more prototypical format. For example, 4 + __ = 9, can be mentally rotated to generate the equation __ = 9 − 4. However, this mental manipulation would require a relatively advanced understanding of calculation rules, that is, a plus becomes a minus when it is moved across the equals sign. Alternatively, children may use spatial visualizations to represent these equations pictorially. This equation could be solved by visualizing 4 blocks in one group and 9 blocks in another, and counting the difference between the groups (Lourenco et al., 2018). It is noteworthy that this study found no significant difference between explicit and implicit instruction on this task in contrast to Hawes et al. (2015) who did not find gains on missing term problems following implicit mental rotation training. This highlights other factors, such as participant engagement during training, as possible explanations for the results reported by Hawes et al. (2015). Another explanation for the differences reported between studies is that in this study and in Cheng and Mix (2014), a part‐whole type mental rotation training was used (participants had to rotate an object and combine it with another object or picture to create a whole) which may have acted as an analog for children when solving missing term problems.

For the Number Line Estimation Task, a significant reduction in error was reported for children who completed spatial scaling training. This far transfer of gains from spatial scaling to number line estimation may be explained by the fact that both tasks require proportional reasoning. If a child was asked to place the number 27 on a number line ranging from 0 to 100, they might reason that 27 is close to 25, which is one quarter of 100. By accurately dividing the number line into quarters, a child could place the number 27 with relatively high accuracy (Newcombe et al., 2015, 2018; Rouder & Geary, 2014). Proportional reasoning is also required when comparing two spaces of different sizes (Newcombe et al., 2018). Alternatively, the Mental Number Line may be responsible for associations between spatial scaling and number line estimation. This concept outlines that numbers are represented spatially in the brain with smaller numbers on the left and larger numbers on the right (Barsalou, 2008; Lakoff & Núñez, 2000). Children may scale between a mental number line and the number line presented in Number Line Estimation Tasks (see Dehaene, 1997; Fischer, 2003). Whilst spatial scaling has been associated with number line estimation in a number of studies (e.g. Gilligan et al., 2018; Mix et al., 2016), this is the first to show that spatial scaling training leads to improvements in number line estimation. To note, an unexpected increase in error was reported following control training. This may be attributable to fatigue or boredom with the task at Time 2. Further investigation is needed to understand this effect.

Performance on the Geometry Task differed across item types. Gains on Geometry Symmetry Items were reported across time, but no effects of training mode or training type were found. Thus, effects in the experimental training conditions did not differ from those in the control conditions. This suggests significant practice effects for this task. In contrast, there was far transfer of training gains from both mental rotation and spatial scaling, to Geometry Shape Items. From a theoretical perspective, children might use spatial visualization (also used in Mental Rotation Tasks) to picture and rotate the shapes presented to count the number of sides (faces) on the shape. Improved spatial scaling skills may have enabled participants to better use proportional reasoning to answer shape items. Instead of counting each individual side (face), participants may have first, segmented the shapes presented (all of which were symmetrical) into halves or thirds, then counted the sides (faces) in a single segment, and finally multiplied this to account for all segments.

*Explicit versus implicit instruction*: For Geometry Shape Items there was a main effect of training mode. Gains were reported following implicit but not explicit instruction. For this task participants were asked to count the number of sides (faces) on a shape. Errors can easily be made on this task by counting the same side (face) twice or by forgetting where on the shape you started counting. As implicit training required participants to carefully select responses and revise incorrect responses, this may have increased the likelihood of participants going back over answers on the Geometry Task, which may in turn have increased accuracy.

For all other measures, there were no main or interaction effects reported for training mode (explicit vs. implicit instruction). This suggests that explicit and implicit spatial instruction are largely similar in eliciting near, intermediate and far transfer of gains. As outlined in the introduction, the efficacy of explicit instruction in this study, maybe be due to the fact that the instructional videos used provide a model of successful task performance, allowing children to acquire new task strategies through observational learning. For implicit instruction, the results of this study suggests that practice with feedback also leads to performance gains. However, here we propose that feedback is a key element of this training type. It may be argued that participants in the control groups in this study completed task practice on account of their completion of the Time 1 task battery. However, these participants did not have significant gains on any measures. This suggests that task practice alone is insufficient to elicit gains, and that the feedback provided in implicit instruction is a key component driving the effectiveness of this training. Taken together, the findings that both explicit and implicit instruction elicit similar gains have practical importance in the classroom. The delivery of instructional videos in a group context offers an easily implementable method of improving spatial thinking that does not require one‐to‐one student interaction or advanced IT facilities (such as a laptop for every student). This mode of instruction offers a feasible, cost‐effective way of spatializing the primary school classroom.

### 4.1 Motivational factors

This study is the first to explore the efficacy of spatial training while controlling for motivational factors. Here we demonstrated that neither participant expectations of, nor engagement with, training can explain the gains reported following spatial training. First, there were no significant differences in participants' expectations of training across different training modes or training types. The similarities in expectations show that differences in expectations of training cannot explain the performance gains reported following training. This increases the reliability of the causal inferences made (Boot et al., 2013). Second, the performance gains reported following training cannot be attributed to engagement with training alone. For explicit training, there were no differences in reported engagement levels between training types. For implicit training, there was significantly higher engagement for participants in the control group compared to those who completed spatial scaling training. Participants who received implicit spatial scaling instruction completed additional trials of the Spatial Scaling Task that they had previously completed at Time 1. However, for the control group the reading task completed was new, that is, not completed at Time 1, and therefore may have been more engaging. Although a significant difference in engagement was found for implicit instruction, the direction of the difference shows that the performance gains reported for spatial and mathematics skills persisted despite the fact that control training may have been more engaging. Taken together, as control training did not lead to gains on any of the outcome measures, levels of engagement did not superficially align with training effects.

### 4.2 Implications, future directions and limitations

**Figure 21:**
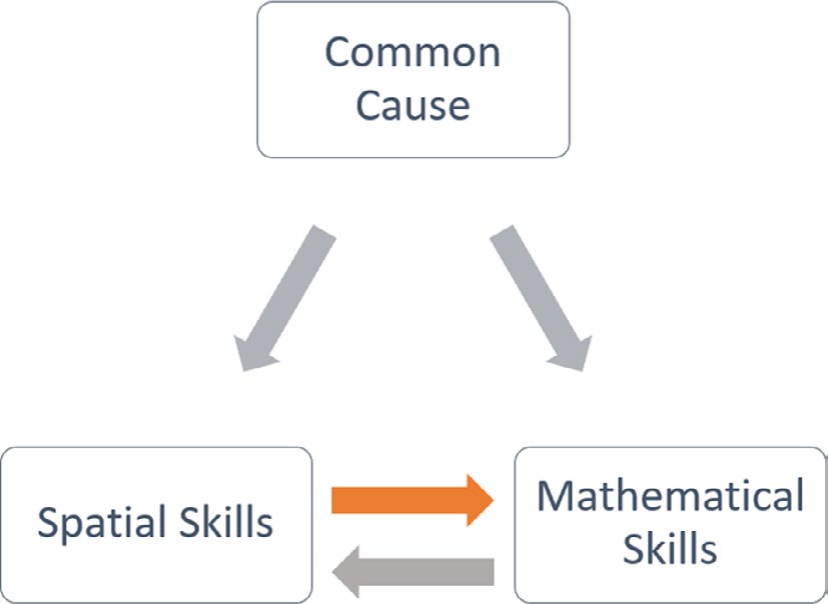
Causal relationship between spatial and mathematical thinking. Note: Established and speculative causal relations are shown in orange and grey respectively

This study provides some of the first evidence that the association between spatial and mathematical performance reflects a causal influence of spatial ability on mathematics performance. This causal relationship between spatial skills and mathematics can be inferred because a manipulation in one variable (spatial skill) led to changes in the other variable (mathematics skill; Pearl, 2000). The findings determine that the observed correlations between spatial and mathematical thinking cannot solely be explained by a common cause acting on both variables, for example, genetic influence, IQ, language skills or other cognitive skills such as WM. As shown in Figure 21, without a direct cause between spatial and mathematical thinking, intervening on spatial skills would not lead to changes in mathematical outcomes. Thus, while a common cause such as a general cognitive factor or neural features may also exist between spatial and mathematical thinking (Oberauer, 2016), this study identified a specific, direct causal effect of spatial skills on mathematics performance. Furthermore, these findings do not preclude a causal role of mathematical thinking on spatial skills, that is, a bidirectional relationship (feedback loop) may exist between spatial and mathematical thinking. From a practical perspective, finding novel methods of improving mathematical thinking in children is an educational priority (National Audit Office UK, 2018) and this study aimed to determine the causal effect of spatial skills on mathematics. However, to establish whether a bidirectional relationship exists between spatial and mathematics skills, future research is needed investigating the effects of training mathematics skills on spatial performance. In summary, the identification of a causal effect of spatial thinking on mathematics in this study, strengthens arguments for spatializing mathematics teaching as a means of improving mathematics outcomes (Bruce & Hawes, 2015). The instructional videos presented here offer one way of introducing spatial thinking into the classroom. However, further research is needed to explore the optimum dosage of this training and the durability of these training gains.

While most previous spatial training studies are based on mental rotation (or similar spatial tasks; Uttal et al., 2013), this study demonstrates an important role for other spatial sub‐domains, particularly spatial scaling. This study highlights the importance of carefully choosing spatial training targets and suggests that training studies should be closely aligned with findings from cross‐sectional and correlational analyses. Mental rotation and spatial scaling were selected as training targets in this study, as these task specifically relate to mathematics outcomes at 8 years (Gilligan et al., 2017; Mix et al., 2016, 2017). Future studies should explore whether spatial training using age appropriate targets might confer benefits to spatial and mathematics performance in older children, for example by training perspective taking abilities or visuo‐spatial thinking which have been associated with mathematics outcomes at 10 years (Gilligan et al., 2018) and 11 years (Mix et al., 2016, 2017) respectively. Furthermore, there is cross‐sectional evidence that the role of spatial thinking extends beyond mathematics, to other Science, Technology, Engineering and Mathematics (STEM) domains (e.g. Hodgkiss, Gilligan, Tolmie, Thomas, & Farran, 2018; Wai, Lubinski, & Benbow, 2009). Future studies could explore transfer of spatial training gains to other STEM domains.

The results of this study should be interpreted in light of its limitations. First, there was a short interval (0–5 min) between training and post‐testing. Therefore, the training completed in this study may have led to priming of certain strategies for task completion, and not conceptual change. Other studies that have shown that short‐term priming is possible and effective in children. For example, 5 min of spatial priming increases creativity in children aged 6–9 years (Liberman, Polack, Hameiri, & Blumenfeld, 2012), while priming spatial language terms (5 min) improves performance on a spatial relations task at 4 years (Loewenstein & Gentner, 2005). However, even if the findings reported reflect a priming effect, the results of this study have significant practical applications for teachers, given that priming enhanced performance on mathematics performance. Alternatively, transfer of gains from spatial training to mathematical skills may reflect both priming and conceptual change. These two processes are necessarily inter‐linked, as it is not possible to prime a process that you have not yet developed. Taken together, although priming cannot be ruled out, similarly to Cheng and Mix (2014), here we demonstrate shared cognitive processing in the completion of spatial and mathematics tasks, that is subject to modification through training. A second limitation of this study was that the duration of the spatial training delivered was relatively short, and there was no investigation of dosage effects. Furthermore, although far transfer of gains between spatial training and mathematical outcomes was reported, the size of these gains was relatively small. Future research is needed to investigate whether the amount of training delivered influences the size and durability of training gains. However, the findings here demonstrate that even short bouts of spatial training lead to transfer of training gains to mathematics. Importantly, the findings of other studies suggest that there is durability of spatial training gains. Uttal et al. (2013) compared spatial training studies with post‐testing immediately following training, to studies that wait days, weeks or even months until post‐testing. Uttal et al. (2013) found that spatial training gains were durable and that the timing of post‐testing did not significantly influence the size of training gains reported following spatial training.

## 5 CONCLUSIONS

The use of developmentally sensitive, theoretically motivated spatial training targets led to near, intermediate and far transfer of gains to both spatial and mathematical domains at 8 years. Not only do these findings highlight the malleability of spatial skills, they also call attention to spatial ability as one domain in which cognitive training can lead to transfer effects. Explicit and implicit instruction led to similar gains in spatial and mathematical domains (except for geometry items). This emphasizes the potential of explicit instruction as a practical means of eliciting far transfer of spatial training gains in the primary school classroom. It is also advised that the choice of cognitive training should be constrained by an understanding of the underlying cognitive mechanisms of training targets. In this study mental visualization was proposed as an underlying cognitive mechanism for mental rotation training, and proportional reasoning was proposed as an underlying cognitive mechanism for spatial scaling training. The gains reported highlight the importance of choosing task and age sensitive targets for spatial training. In turn, evidence from this training study lays bare the causal contribution of cognitive processes to mathematical cognition that was previously only inferred based on correlational evidence.

## CONFLICT OF INTEREST

The authors have no conflicts of interest to declare.

## Data Availability

The data that support the findings of this study are available from the corresponding author Katie Gilligan (k.gilligan@surrey.ac.uk) upon reasonable request.

## References

[desc12909-bib-0001] Barsalou, L. W. (2008). Grounded cognition. Annual Review of Psychology, 59(1), 617–645. 10.1146/annurev.psych.59.103006.093639 17705682

[desc12909-bib-0002] Boot, W. R. , Simons, D. J. , Stothart, C. , & Stutts, C. (2013). The pervasive problem with placebos in psychology: Why active control groups are not sufficient to rule out placebo effects. Perspectives on Psychological Science, 8(4), 445–454. 10.1177/1745691613491271 26173122

[desc12909-bib-0003] Bruce, C. D. , & Hawes, Z. (2015). The role of 2D and 3D mental rotation in mathematics for young children: What is it? Why does it matter? And what can we do about it? ZDM Mathematics Education, 47(3), 331–343. 10.1007/s11858-014-0637-4

[desc12909-bib-0004] Castro‐Alonso, J. C. , Ayres, P. , & Paas, F. (2014). Learning from observing hands in static and animated versions of non‐manipulative tasks. Learning and Instruction, 34, 11–21. 10.1016/j.learninstruc.2014.07.005

[desc12909-bib-0005] Cheng, Y. L. , & Mix, K. S. (2014). Spatial training improves children's mathematics ability. Journal of Cognition and Development, 15(1), 2–11. 10.1080/15248372.2012.725186

[desc12909-bib-0006] Cohen, C. A. , & Hegarty, M. (2014). Visualizing cross sections: Training spatial thinking using interactive animations and virtual objects. Learning and Individual Differences, 33, 63–71. 10.1016/j.lindif.2014.04.002

[desc12909-bib-0007] Cohen, D. (1988). Statistical power analysis for the behavioural sciences (2nd ed.). Hillsdale, NJ: Lawrence Earlbaum Associates.

[desc12909-bib-0008] Dehaene, S. (1997). The number sense: How the mind creates mathematics. New York, NY: Oxford University Press.

[desc12909-bib-0009] Department of Education . (2013). Mathematics programmes of study: Key stages 1 and 2 National curriculum in England. Retrieved from https://www.gov.uk/government/publications/national-curriculum-in-england-mathematics-programmes-of-study

[desc12909-bib-0010] Dweck, C. S. (2000). Self‐theories: Their role in motivation, personality, and development. Philadelphia, PA: Psychology Press.

[desc12909-bib-0011] Fias, W. , & Bonato, M. (2018). Chapter 12 – Which space for numbers? In Henik A. & Fias W. (Eds.), Heterogeneity of function in numerical cognition (pp. 233–242). Academic Press Retrieved from http://www.sciencedirect.com/science/article/pii/B9780128115299000121

[desc12909-bib-0012] Field, A. (2013). Discovering statistics using IBM SPSS Statistics: And sex and drugs and rock ‘n’ roll (4th edn). London: Sage.

[desc12909-bib-0014] Fischer, M. (2003). Spatial representations in number processing–evidence from a pointing task. Visual Cognition, 10(4), 493–508. 10.1080/13506280244000186

[desc12909-bib-0015] Foroughi, C. K. , Monfort, S. S. , Paczynski, M. , McKnight, P. E. , & Greenwood, P. M. (2016). Placebo effects in cognitive training. Proceedings of the National Academy of Sciences of the United States of America, 113(27), 7470–7474. 10.1073/pnas.1601243113 27325761 PMC4941515

[desc12909-bib-0016] Frick, A. (2019). Spatial transformation abilities and their relation to later mathematics performance. Psychological Research Psychologische Forschung, 83(7), 1465–1484. 10.1007/s00426-018-1008-5 29637258

[desc12909-bib-0017] Frick, A. , & Newcombe, N. S. (2012). Getting the big picture: Development of spatial scaling abilities. Cognitive Development, 27(3), 270–282. 10.1016/j.cogdev.2012.05.004

[desc12909-bib-0018] Gallagher‐Mitchell, T. , Romero‐Rivas, C. , Rodriguez‐Cuadrado, S. , & Dackermann, T. (2017). Age, cross‐linguistic, and modality effects on children's number line estimation. Presented at the European Society for Cognitive Psychology Conference, Potsdam, Germany.

[desc12909-bib-0019] Gallagher‐Mitchell, T. , Simms, V. , & Litchfield, D. (2018). Learning from where “eye” remotely look or point: Impact on number line estimation error in adults. Quarterly Journal of Experimental Psychology, 71(7), 1526–1534. 10.1080/17470218.2017.1335335 28540753

[desc12909-bib-0020] Gilligan, K. A. , Flouri, E. , & Farran, E. K. (2017). The contribution of spatial ability to mathematics achievement in middle childhood. Journal of Experimental Child Psychology, 163, 107–125. 10.1016/j.jecp.2017.04.016 28753435

[desc12909-bib-0021] Gilligan, K. A. , Hodgkiss, A. , Thomas, M. S. C. , & Farran, E. K. (2018). The developmental relations between spatial cognition and mathematics in primary school children. Developmental Science, 22, e12786. 10.1111/desc.12786 30548725

[desc12909-bib-0022] Green, S. C. , Bavelier, D. , Kramer, A. , Vinogradov, S. , Ansorge, U. , Ball, K. , & Witt, C. (2019). Improving methodological standards in behavioral interventions for cognitive enhancement. Journal of Cognitive Enhancement, 3(1), 2–29. 10.1007/s41465-018-0115-y

[desc12909-bib-0023] Hawes, Z. , Moss, J. , Caswell, B. , Naqvi, S. , & MacKinnon, S. (2017). Enhancing children's spatial and numerical skills through a dynamic spatial approach to early geometry instruction: Effects of a 32‐week intervention. Cognition and Instruction, 35(3), 236–264. 10.1080/07370008.2017.1323902

[desc12909-bib-0024] Hawes, Z. , Moss, J. , Caswell, B. , & Poliszczuk, D. (2015). Effects of mental rotation training on children's spatial and mathematics performance: A randomized controlled study. Trends in Neuroscience and Education, 4(3), 60–68. 10.1016/j.tine.2015.05.001

[desc12909-bib-0025] Hodgkiss, A. , Gilligan, K. A. , Tolmie, A. K. , Thomas, M. S. C. , & Farran, E. K. (2018). Spatial cognition and science achievement: The contribution of intrinsic and extrinsic spatial skills from 7 to 11 years. British Journal of Educational Psychology, 88(4), 675–697. 10.1111/bjep.12211 29359476 PMC6283002

[desc12909-bib-0026] Jaeggi, S. M. , Buschkuehl, M. , Shah, P. , & Jonides, J. (2014). The role of individual differences in cognitive training and transfer. Memory & Cognition, 42(3), 464–480. 10.3758/s13421-013-0364-z 24081919

[desc12909-bib-0027] Kirschner, P. A. , Sweller, J. , & Clark, R. E. (2006). Why minimal guidance during instruction does not work: An analysis of the failure of constructivist, discovery, problem‐based, experiential, and inquiry‐based teaching. Educational Psychologist, 41(2), 75–86. 10.1207/s15326985ep4102_1

[desc12909-bib-0028] Lakoff, G. , & Núñez, R. (2000). Where mathematics comes from: How the embodies brings mathematics into being. New York, NY: Basic Books.

[desc12909-bib-0029] Liberman, N. , Polack, O. , Hameiri, B. , & Blumenfeld, M. (2012). Priming of spatial distance enhances children's creative performance. Journal of Experimental Child Psychology, 111(4), 663–670. 10.1016/j.jecp.2011.09.007 22078295

[desc12909-bib-0030] Loewenstein, J. , & Gentner, D. (2005). Relational language and the development of relational mapping. Cognitive Psychology, 50, 315–353. 10.1016/j.cogpsych.2004.09.004 15893523

[desc12909-bib-0031] Lourenco, S. F. , Cheung, C.‐N. , & Aulet, L. S. (2018). Chapter 10 – Is visuospatial reasoning related to early mathematical development? A critical review In Henik A. & Fias W. (Eds.), Heterogeneity of function in numerical cognition (pp. 177–210). Academic Press Retrieved from http://www.sciencedirect.com/science/article/pii/B9780128115299000108

[desc12909-bib-0032] Lowrie, T. , Logan, T. , & Ramful, A. (2017). Visuospatial training improves elementary students' mathematics performance. British Journal of Educational Psychology, 87(2), 170–186. 10.1111/bjep.12142 28097646

[desc12909-bib-0033] Mix, K. S. , Levine, S. C. , Cheng, Y.‐L. , Young, C. J. , Hambrick, D. Z. , & Konstantopoulos, S. (2017). The latent structure of spatial skills and mathematics: A replication of the two‐factor model. Journal of Cognition and Development, 18(4), 465–492. 10.1080/15248372.2017.1346658

[desc12909-bib-0034] Mix, K. S. , Levine, S. C. , Cheng, Y.‐L. , Young, C. , Hambrick, D. Z. , Ping, R. , & Konstantopoulos, S. (2016). Separate but correlated: The latent structure of space and mathematics across development. Journal of Experimental Psychology: General, 145(9), 1206–1227. 10.1037/xge0000182 27560854

[desc12909-bib-0035] Möhring, W. , Newcombe, N. S. , & Frick, A. (2016). Using mental transformation strategies for spatial scaling: Evidence from a discrimination task. Journal of Experimental Psychology: Learning, Memory, and Cognition, 42(9), 1473–1479. 10.1037/xlm0000240 26844579

[desc12909-bib-0036] National Audit Office UK . (2018). Delivering STEM (science, technology, engineering and mathematics) skills for the economy. London: Department for Business, Energy & Industrial Strategy Department for Education.

[desc12909-bib-0037] Neuburger, S. , Jansen, P. , Heil, M. , & Quaiser‐Pohl, C. (2011). Gender differences in pre‐adolescents' mental‐rotation performance: Do they depend on grade and stimulus type? Personality and Individual Differences, 50(8), 1238–1242. 10.1016/j.paid.2011.02.017

[desc12909-bib-0038] Newcombe, N. S. , Levine, S. C. , & Mix, K. S. (2015). Thinking about quantity: The intertwined development of spatial and numerical cognition. Wiley Interdisciplinary Reviews: Cognitive Science, 6(6), 491–505. 10.1002/wcs.1369 26415916

[desc12909-bib-0039] Newcombe, N. S. , Möhring, W. , & Frick, A. (2018). Chapter 9 – How big is many? Development of spatial and numerical magnitude understanding In Henik A. & Fias W. (Eds.), Heterogeneity of function in numerical cognition (pp. 157–176). Academic Press Retrieved from http://www.sciencedirect.com/science/article/pii/B9780128115299000091

[desc12909-bib-0040] Oberauer, K. (2016). Parameters, not processes, explain general intelligence. Psychological Inquiry, 27(3), 231–235. 10.1080/1047840X.2016.1181999

[desc12909-bib-0041] Paas, F. , & Sweller, J. (2012). An evolutionary upgrade of cognitive load theory: Using the human motor system and collaboration to support the learning of complex cognitive tasks. Educational Psychology Review, 24(1), 27–45. 10.1007/s10648-011-9179-2

[desc12909-bib-0042] Pearl, J. (2000). Causality: Models, reasoning and inference (Vol. 29). Cambridge: MIT Press.

[desc12909-bib-0043] Rizzolatti, G. , & Sinigaglia, C. (2010). The functional role of the parieto‐frontal mirror circuit: Interpretations and misinterpretations. Nature Reviews Neuroscience, 11(4), 264–274. 10.1038/nrn2805 20216547

[desc12909-bib-0044] Rouder, J. N. , & Geary, D. C. (2014). Children's cognitive representation of the mathematical number line. Developmental Science, 17(4), 525–536. 10.1111/desc.12166 24796511 PMC4439402

[desc12909-bib-0045] Siegler, R. S. , & Opfer, J. E. (2003). The development of numerical estimation: Evidence for multiple representations of numerical quantity. Psychological Science, 14(3), 237–243. 10.1111/1467-9280.02438 12741747

[desc12909-bib-0046] Simms, V. , Clayton, S. , Cragg, L. , Gilmore, C. , & Johnson, S. (2016). Explaining the relationship between number line estimation and mathematical achievement: The role of visuomotor integration and visuospatial skills. Journal of Experimental Child Psychology, 145, 22–33. 10.1016/j.jecp.2015.12.004 26773209

[desc12909-bib-0047] Tettamanti, M. , Buccino, G. , Saccuman, M. C. , Gallese, V. , Danna, M. , Scifo, P. , … Perani, D. (2005). Listening to action‐related sentences activates fronto‐parietal motor circuits. Journal of Cognitive Neuroscience, 17(2), 273–281. 10.1162/0898929053124965 15811239

[desc12909-bib-0048] Uttal, D. H. , Meadow, N. G. , Tipton, E. , Hand, L. L. , Alden, A. R. , Warren, C. , & Newcombe, N. S. (2013). The malleability of spatial skills: A meta‐analysis of training studies. Psychological Bulletin, 139(2), 352–402. 10.1037/a0028446 22663761

[desc12909-bib-0049] Verdine, B. N. , Golinkoff, R. M. , Hirsh‐Pasek, K. , Newcombe, N. S. , Filipowicz, A. T. , & Chang, A. (2014). Deconstructing building blocks: Preschoolers' spatial assembly performance relates to early mathematical skills. Child Development, 85(3), 1062–1076. 10.1111/cdev.12165 24112041 PMC3962809

[desc12909-bib-0050] Wai, J. , Lubinski, D. , & Benbow, C. P. (2009). Spatial ability for STEM domains: Aligning over 50 years of cumulative psychological knowledge solidifies its importance. Journal of Educational Psychology, 101(4), 817–835. 10.1037/a0016127

