# Supplementary material for: First demonstration of effective spatial training for near transfer to spatial performance and far transfer to a range of mathematics skills at 8 years
Source: Dev Sci. 2019 Nov 6;23(4):e12909. doi: 10.1111/desc.12909 (PMC7379338; doi:10.1111/desc.12909)
Supplement: Supplementary file 1 [file DESC-23-e12909-s001.pdf]

Supplementary material

Assessing training effects using ANCOVA analysis

As outlined in the results section, one-way ANCOVAs with baseline performance as a covariate were also completed to explore training effects. ANCOVAs were completed for each task in the test battery. Training mode (2 levels: explicit, implicit) and training type (3 levels: mental rotation, spatial scaling, literacy) were included as between participant variables. Post-training scores (Time 2) were included as the dependent variable and pre-training scores (Time 1) were included as a covariate.

Consistent with the MANOVA results reported in the main manuscript, there was a main effect of training type for: the Mental Rotation Task, F (2,236) = 4.96, p = .008, η_p^2 = .040; the Spatial Scaling task, F (2,231) = 12.09, p < .001, η_p^2 = .094; Missing Term Problems, F (2, 208) = 3.85, p = .023, η_p^2 = .036; PAE scores on the Number Line Estimation Task, F (2,236) = 7.29, p = .001, η_p^2 = .058, and; Geometry Shape Items, F (1,218) = 4.91, p = .008, η_p^2 = .043. Consistent with the results reported in the main manuscript, there was no main effect of training type for Geometry Symmetry Items, F (2,212) = 0.55, p = .877, η_p^2 = .005.

To further explore the main effects of training type, bonferroni comparisons were used to compare performance at Time 2 across training types. For the Mental Rotation Task, pairwise comparisons demonstrated that those who completed mental rotation training had significantly higher scores at Time 2 than those who completed control training (p = .006). The mental rotation training group also had higher scores than those who completed spatial scaling training, although this difference was not significant (p = .305). There was no significant difference in performance between those who completed spatial scaling and control training (p = .397). For the Spatial Scaling Task, pairwise comparisons indicated that performance at Time 2 was significantly higher for those who completed spatial scaling training compared to those who completed mental rotation training (p <.001) or control training (p <.001). There was no significant difference in performance between those who completed mental rotation and control training (p = 1.00).

For Missing Term Problems, pairwise comparisons indicated that those who completed mental rotation training had higher performance scores than those who completed spatial

scaling training (p = .032) or control training (p = .085). There was no significant difference in performance between those who completed spatial scaling and control training (p = 1.00). For PAE scores on the Number Line Estimation Task, error scores were significantly lower after spatial scaling training compared to mental rotation training (p = .036) and control training (p < .001). Error scores were lower, although not significantly, for the mental rotation training group compared to the control training group (p = .085). Finally, for the Geometry Shape Items, performance was significantly higher following both mental rotation training (p = .024) and spatial scaling training (p = .019) compared to control training. There was no significant difference in performance between the mental rotation and spatial scaling training groups (p = 1.00)

For training mode, there was a significant main effect for Geometry Shape Items, F (2,212) = 0.55, p = .877, η_p^2 = .005. This favoured implicit instruction. No other main effects of training mode were found for: the Mental Rotation Task, F (1,236) = 0.01, p = .969, η_p^2 = .001; the Spatial Scaling task, F (1,231) = 2.28, p = .133, η_p^2 = .010; Missing Term Problems, F (1, 208) = 2.43, p = .120, η_p^2 = .012, Geometry Symmetry Items, F (2,212) = 0.15, p = .701, η_p^2 = .001, and; PAE scores on the Number Line Estimation Task, F (1,236) = 2.99, p = .085, η_p^2 = .013. There were no significant interactions between training type and training mode for any task (p's > .391; η_p^2's < .008).
